# Defeating a superbug: A breakthrough in vaccine design against multidrug-resistant *Pseudomonas aeruginosa* using reverse vaccinology

**DOI:** 10.1371/journal.pone.0289609

**Published:** 2023-08-03

**Authors:** Sepideh Fereshteh, Fatemeh Haririzadeh Jouriani, Narjes Noori Goodarzi, Mahdi Torkamaneh, Behnoush Khasheii, Farzad Badmasti

**Affiliations:** 1 Department of Bacteriology, Pasteur Institute of Iran, Tehran, Iran; 2 Department of Pathobiology, School of Public Health, Tehran University of Medical Sciences, Tehran, Iran; 3 Department of Pathobiology, Faculty of Veterinary Science, Bu-Ali Sina University, Hamedan, Iran; The Islamia University of Bahawalpur Pakistan, PAKISTAN

## Abstract

**Background:**

Multidrug-resistant *Pseudomonas aeruginosa* has become a major cause of severe infections. Due to the lack of approved vaccines, this study has presented putative vaccine candidates against it.

**Methods:**

*P*. *aeruginosa* 24Pae112 as a reference strain was retrieved from GenBank database. The surface-exposed, antigenic, non-allergenic, and non-homologous human proteins were selected. The conserved domains of selected proteins were evaluated, and the prevalence of proteins was assessed among 395 genomes. Next, linear and conformational B-cell epitopes, and human MHC II binding sites were determined. Finally, five conserved and highly antigenic B-cell epitopes from OMPs were implanted on the three platforms as multi-epitope vaccines, including FliC, the bacteriophage T7 tail, and the cell wall-associated transporter proteins. The immunoreactivity was investigated using molecular docking and immune simulation. Furthermore, molecular dynamics simulation was done to refine the chimeric cell-wall-associated transporter-TLR4 complex as the best interaction.

**Results:**

Among 6494 total proteins of *P*. *aeruginosa* 24Pae112, 16 proteins (seven OMPs and nine secreted) were ideal according to the defined criteria. These proteins had a molecular weight of 110 kDa and were prevalent in ≥ 75% of *P*. *aeruginosa* genomes. Among the presented multi-epitope vaccines, the chimeric cell-wall-associated transporter had the strongest interaction with TLR4. Moreover, the immune simulation response revealed that the bacteriophage T7 tail chimeric protein had the strongest ability to stimulate the immune system. In addition, molecular docking and molecular dynamic simulation indicated the proper and stable interactions between the chimeric cell-wall-associated transporter and TLR4.

**Conclusion:**

This study proposed 16 shortlisted proteins as promising immunogenic targets. Two novel platforms (*e*.*g*. cell-wall-associated transporter and bacteriophage T7 tail proteins) for designing of multi-epitope vaccines (MEVs), showed the better performance compared to FliC. In our future studies, these two MEVs will receive more scrutiny to evaluate their immunoreactivity.

## 1. Introduction

*Pseudomonas aeruginosa* is a ubiquitous Gram-negative bacterium that belongs to the *Pseudomonadaceae* family and can survive in a variety of environments, including soil and water. Indeed, it can be easily found in almost any human- or animal-influenced habitat [1]. Studies have shown that in healthcare settings, this bacterium causes fatal nosocomial infections such as pneumonia, urinary tract infections, wound infections, and septicemia, especially in immunocompromised individuals [2, 3]. In addition, *P*. *aeruginosa* is responsible for more than 5% of infectious exacerbations in patients with chronic obstructive pulmonary disease (COPD) and has been associated with increased mortality in these patients [4]. In 2017, carbapenem-resistant *P*. *aeruginosa* was recognized as one of the most life-threatening bacteria and listed by the World Health Organization (WHO) as a priority pathogen for the research and development of new ways to overcome it [5].

The overuse of antibiotics during treatment accelerates the development of multidrug-resistant *P*. *aeruginosa* strains, leading to the ineffectiveness of antibiotic therapy against this microorganism [6]. Therefore, there is an urgent need to discover new strategies for the prevention of severe *P*. *aeruginosa* infections. To date, different approaches, such as secondary metabolites, nanomaterials, and vaccine development, have been suggested to find a suitable alternative to the currently used antibiotics against this pathogen. However, because of ongoing problems such as growing antibiotic resistance and population aging, vaccination could be more desirable and cost-effective [7, 8].

Currently, numerous vaccine development methods are being tested against this superbug. For example, whole-cell vaccines [9], killed and attenuated live vaccines [10, 11], outer membrane vesicles (OMVs) [12], outer membrane complexes (OMCs) [13], flagella [14], pilins [7], and glycol-conjugate vaccines [15] have been proposed as promising vaccination approaches against *P*. *aeruginosa*. Although many of these approaches have been tested experimentally in preclinical trials, few of them have reached the clinical phase, and none of them has been approved for marketing [7]. Therefore, in the absence of an effective vaccine, efforts are continuing.

Present immunization strategies mainly concentrate on outer membrane proteins (OMPs) as desirable subunit vaccines since there is no risk of the pathogen reverting to its virulent form and there are few adverse effects compared to live attenuated, or killed whole-cell vaccines [7]. Moreover, on the surface of bacterial cells, OMPs play a key role in bacterial physiology and pathogenesis; therefore, they are considered prime targets for vaccine development [16]. In this regard, reverse vaccinology (RV) allows researchers to use the whole bacterial genome sequence to find immunogenic and surface-exposed proteins and study them from different aspects such as antigenicity, allergenicity, and similarity to the human proteome [17, 18]. As a result, a new generation of vaccines can be developed based on antigens that were previously not detected or even ignored. For example, RV has been successfully used to produce the 4CMenB vaccine (Bexsero, GSK) as the first approved vaccine against *Neisseria meningitidis* serogroup B (MenB), which is currently licensed in several countries [19, 20].

Taking into account the aforementioned, the present study aimed to investigate the whole genome sequence of *P*. *aeruginosa* 24Pae112 to introduce novel and putative vaccine candidates. Also the design of new multi-epitope vaccines against this bacterium can be considered as promising subunit vaccines. The obtained results from this study provide new insights into vaccine development not only against *P*. *aeruginosa* but also against other Gram-negative bacteria.

## 2. Materials and methods

### 2.1. Consecutive analyses

#### 2.1.1 Genomes sequence retrieval

In the first step, 395 completed genomes of *P*. *aeruginosa* were retrieved from the GenBank database (https://www.ncbi.nlm.nih.gov/genbank/) and extracted to the proteome using CLC Genomics Workbench Software (Qiagen, Hilden, Germany) [21] to perform core/pan-genome analysis using BPGA (Bacterial Pan Genome Analysis Tool) software [22]. After genome evaluation, *P*. *aeruginosa* 24Pae112 (accession number: NZ_CP053028.1), which belongs to ST235, was selected as a reference strain. *P*. *aeruginosa* ST235 is spread around the world, possibly due to selective pressure from fluoroquinolones, and became easily resistant to aminoglycosides, beta-lactams, and carbapenems through mutation and acquisition of resistance elements [23].

#### 2.1.2 Prediction of subcellular localization

All proteins were uploaded to the PSORTb database (https://www.psort.org/psortb/) to predict their subcellular localization [24]. In this step, extracellular, secreted, and surface-exposed proteins were considered. The results were confirmed using the TMHMM Server v. 2.0 web tool (https://services.healthtech.dtu.dk/service.php?TMHMM-2.0) to identify surface-exposed regions [25].

#### 2.1.3 Antigenicity and allergenicity determination

The antigenicity of putative immunogenic proteins was predicted using the VaxiJen online tool (http://www.ddgpharmfac.net/vaxijen/VaxiJen/VaxiJen.html) with a cut-off value of ≥ 0.4 and ANTIGENpro (https://scratch.proteomics.ics.uci.edu/) [26, 27]. On the other hand, the allergenicity of the putative immunogenic proteins was predicted using AlgPred 2.0 (http://crdd.osdd.net/raghava/algpred/) with a cut-off value of ≥ 0.3 and AllergenFP (https://www.ddg-pharmfac.net/AllergenFP/) [28, 29].

#### 2.1.4 Homology analysis of immunogenic targets against the human proteome

All selected proteins were analyzed to determine their sequence similarity to the human proteome (*Humo sapiens*, Taxid: 9606) using the PSI-BLAST tool in the BLASTp database (https://blast.ncbi.nlm.nih.gov/Blast.cgi?SIDE=protein) [30]. Proteins showing any similarity to the proteome of the host were excluded from the study.

#### 2.1.5 Functional class determination and molecular weight estimation

First, the functional class of the selected proteins was determined using the VICMpred database (https://webs.iiitd.edu.in/raghava/vicmpred/submission.html) [31]. Then, the number of amino acids and molecular weights were determined using the Expasy ProtParam server (https://web.expasy.org/protparam/) [32].

#### 2.1.6 Protein domain search

Conserved Domain Database, CDD (https://www.ncbi.nlm.nih.gov/Structure/cdd/cdd.shtml) and EggNOG (http://eggnog5.embl.de/#/app/home) were used to find the protein domains. CDD is a part of the NCBI’s Entrez query and provides annotation of protein sequences with the position of the conserved domain [33]. EggNOG is an annotated orthology resource according to 5090 organisms and 2502 viruses [34].

#### 2.1.7 Prevalence of putative immunogenic targets among *P*. *aeruginosa* genomes dataset

Whole genome sequences of 395 *P*. *aeruginosa* strains were checked using BPGA software, and the prevalence matrix as software output was used to determine the prevalence of each protein [22]. In this step, the cut-off value of ≥ 75% was considered.

### 2.2. Immunoinformatics analyses

#### 2.2.1 Determination of linear B-cell epitopes and human MHC II binding sites

The BepiPred-2.0 tool (https://services.healthtech.dtu.dk/service.php?BepiPred-2.0) was used to identify linear B-cell epitopes of all previously selected proteins with a threshold of ≥ 0.6 [35]. The B-cell epitope ratio (number of amino acids of all epitopes divided by the total amino acids of each protein) was calculated for each protein, and proteins with a ratio above the average were selected. In the next step, TepiTool (http://tools.iedb.org/tepitool/), an IEDB resource, was used to predict human MHC II binding sites with a cut-off of the top 5% of peptides [36]. The ratios of MHC II binding sites (the number of MHC II binding sites divided by the total amino acids of each protein) were calculated.

#### 2.2.2 Quartile scoring method

The selected proteins were analyzed by the quartile scoring method using three different indicators, including antigenicity, linear B-cell epitope, and MHC II binding site ratios. The sum of all scores for each protein was considered the final score [21].

#### 2.2.3 Tertiary structure prediction and characterization of conformational B-cell epitopes

The tertiary structure (3D) of the putative immunogenic proteins was predicted using the Robetta tool (https://robetta.bakerlab.org/) [37]. The quality of the 3D model was checked using the ProSA web server (https://prosa.services.came.sbg.ac.at/prosa.ph) [38]. This server shows the potential errors in the 3D model. The energetically refused and permitted regions were predicted using Ramachandran plots at the Zlab Ramachandran Plot Server (https://zlab.umassmed.edu/bu/rama/index.pl) [39]. ElliPro (http://tools.iedb.org/ellipro/) was used to identify the conformational B-cell epitopes with a threshold of ≥ 0.8 [40]. The predicted conformational B-cell epitopes were displayed on the surface of each protein in different colors using the Jmol software [41].

#### 2.2.4 Protein-protein interaction based on the STRING database

In this part, we used the STRING database (https://string-db.org/) to investigate the interactions of two Hypothetical proteins with other proteins of *P*. *aeruginosa* to estimate their function. In molecular biology, STRING is a biological database and web resource for known and predicted protein-protein interactions [42].

### 2.3. Construction of multi-epitope vaccines

#### 2.3.1 Selecting antigenic and conserved linear B-cell epitopes

Linear B-cell epitopes, located on the extracellular loops of seven OMPs selected from previous steps, were predicted using the BepiPred database with a cut-off value of ≥ 0.6. Additionally, the conservation of linear B-cell epitopes was determined using the ConSurf web tool (https://consurf.tau.ac.il/consurf_index.php) [43].

#### 2.3.2 Implantation of conserved linear B-cell epitopes on platforms

Three multi-epitope-based vaccines were generated using three different platforms obtained from *P*. *aeruginosa* strain 24Pae112, including FliC (WP_124119965.1), bacteriophage T7 tail protein (WP_166796845.1), and cell-wall-associated transporter (WP_024947839.1). The tertiary (3D) structures of these chimeras were modeled using the Robetta web tool. The 3D structures were validated by the ProSA web server (https://prosa.services.came.sbg.ac.at/prosa.php) and Ramachandran Plot (https://zlab.umassmed.edu/bu/rama/).

#### 2.3.3 Molecular dockings and immune simulations

Molecular dockings and the binding affinities of three multi-epitope vaccines to human TLR1 (PDB: 2Z7X), TLR2 (PDB: 2Z7X), and TLR4 (PDB: 3FXI) were assessed with pyDockWEB (https://life.bsc.es/pid/pydockweb/default/index). In addition, C-ImmSim (https://kraken.iac.rm.cnr.it/C-IMMSIM/index.php) was used to predict the simulation of the immunoreactivity of multi-epitope vaccines [21]. Finally, PDBsum (http://www.ebi.ac.uk/thornton-srv/databases/pdbsum/) was used to reveal the paired residues of the chimeric cell-wall-associated transporter and TLR4 [44].

#### 2.3.4 Molecular dynamics simulation of the selected vaccine in complex with the immune receptor

Molecular dynamics (MD) simulation was done to refine the chimeric cell-wall-associated transporter-TLR4 complex using the GROMACS version 2018 simulation package. The structure was centered in a dodecahedron box and filled with water using the TIP3 water model. To neutralize the system, some molecules of water were randomly replaced by Cl^-^ or Na^+^. After neutralization, the energy minimization (5000 steps) was done using the steepest descent algorithm. Equilibrating the system was performed under 100 ps NVT at a temperature of 298 K, followed by 100 ps NPT ensembles at a pressure of 1 bar. Electrostatic interactions were calculated by PME, and the LINCS procedure was applied to constrain all bonds connecting hydrogen atoms. The final MD simulation was run for 100 ns with no restraint [45, 46].

## 3. Results

### 3.1. Core/pan-genome analysis and subcellular localization prediction

**Fig 1:**
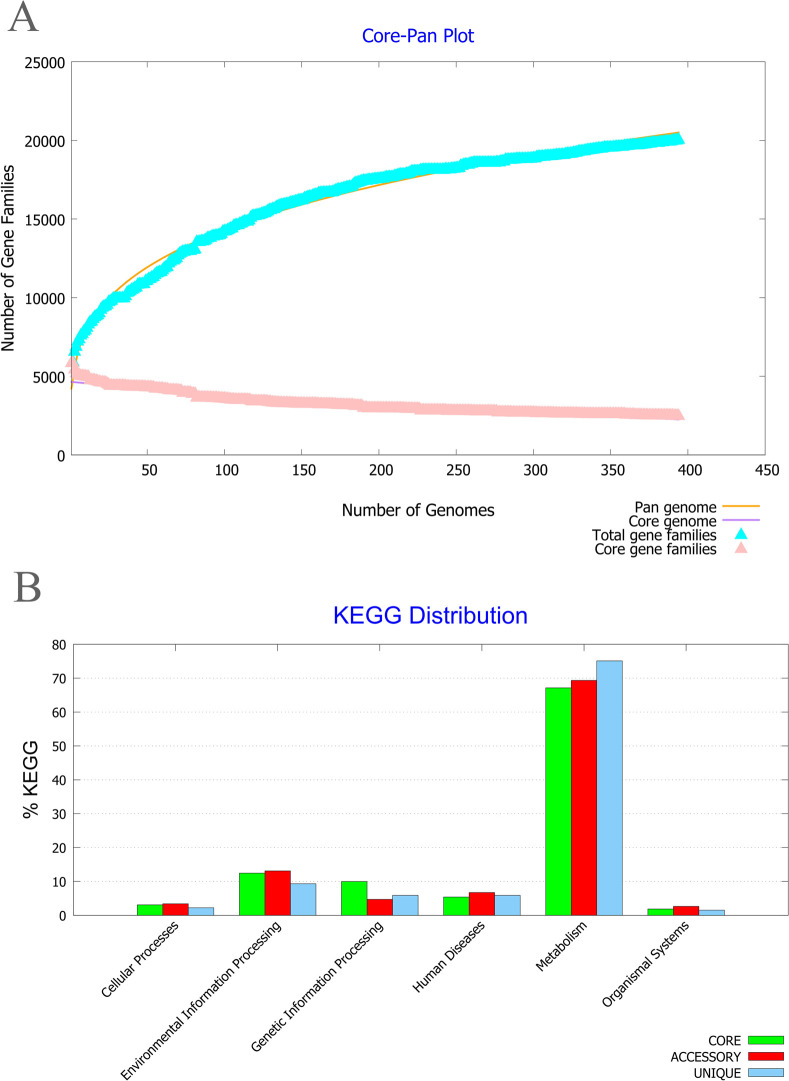
Core-proteome analysis using the BPGA database. **A)** The core/pan plot of 395 *P*. *aeruginosa* strains showed a total of 2547 core proteins. **B)** The KEGG distribution of core, accessory, and unique genes of *P*. *aeruginosa* strains represented that the majority of all three categories are involved in the metabolism of this pathogen.

**Fig 2:**
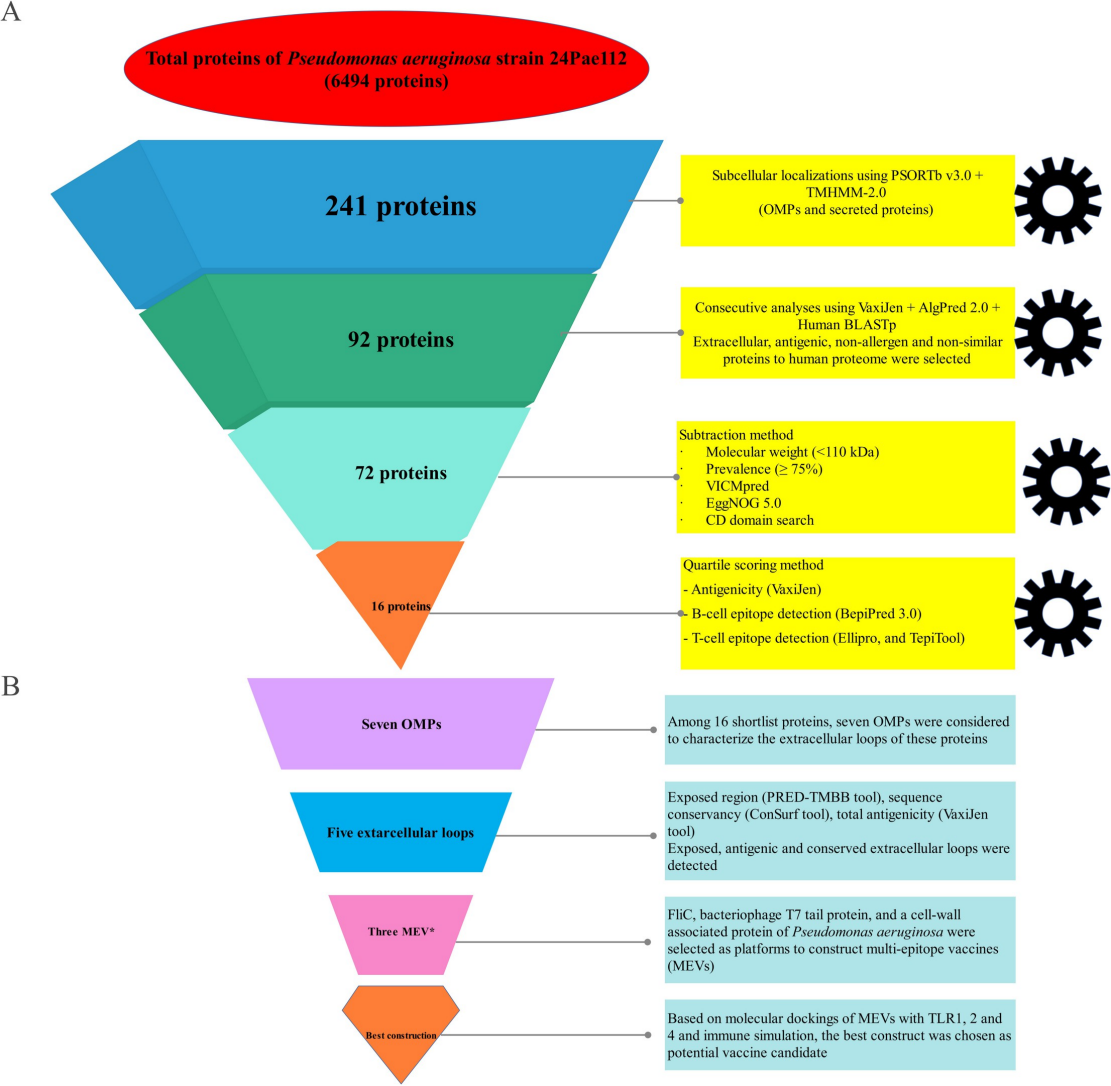
Schematic representation of the selection and validation of new putative immunogenic targets and multi-epitope-based vaccines against *P*. *aeruginosa* strain 24Pae112 using reverse vaccinology approaches and bioinformatics tools. All criteria and thresholds are shown in the flowchart. OMPs, outer membrane proteins; MEVs, multi-epitope vaccines.

The core/pan-genome ratio and B-parameter (0.24) obtained from BPGA analysis on 395 completed genomes of *P*. *aeruginosa* revealed that this bacterium has a wide genome. These results indicated the plasticity of the *P*. *aeruginosa* genome due to many insertion and deletion events. Therefore, using the core proteome was not proper, and we used the whole genome of *P*. *aeruginosa* 24Pae112 instead (Fig 1A). The KEGG (Kyoto Encyclopedia of Genes and Genomes) distribution plot revealed that the majority of core, accessory, and unique genes were involved in the metabolism of this pathogen (Fig 1B). *P*. *aeruginosa* strain 24Pae112 showed that the genome has a total of 6494 proteins. Among them, a total of 241 surface-exposed proteins consisting of 167 OMPs and 74 extracellular proteins were identified, and others were excluded. The workflow for identifying new immunogenic targets and designing multi-epitope vaccines against *P*. *aeruginosa* 24Pae112 has been presented in Fig 2.

### 3.2. Consecutive analysis revealed desirable proteins

Back-to-back analysis of selected proteins from the previous step, revealed that out of 241 proteins, 92 were antigenic, non-allergenic, and non-homologous to human proteins. Therefore, these proteins were selected for further analysis.

### 3.3. Subtraction of the proteins based on molecular weight and prevalence

According to the defined criteria, 72 proteins with a molecular weight < 110 kDa and a prevalence ≥ 75% among *P*. *aeruginosa* genomes were selected. These proteins were involved in different functional classes, including virulence (22 proteins), cellular processes (35 proteins), metabolism (13 proteins), and information and storage (two proteins). See the S1 Table.

### 3.4. Quartile scoring assessment to select shortlisted proteins

**Fig 3:**
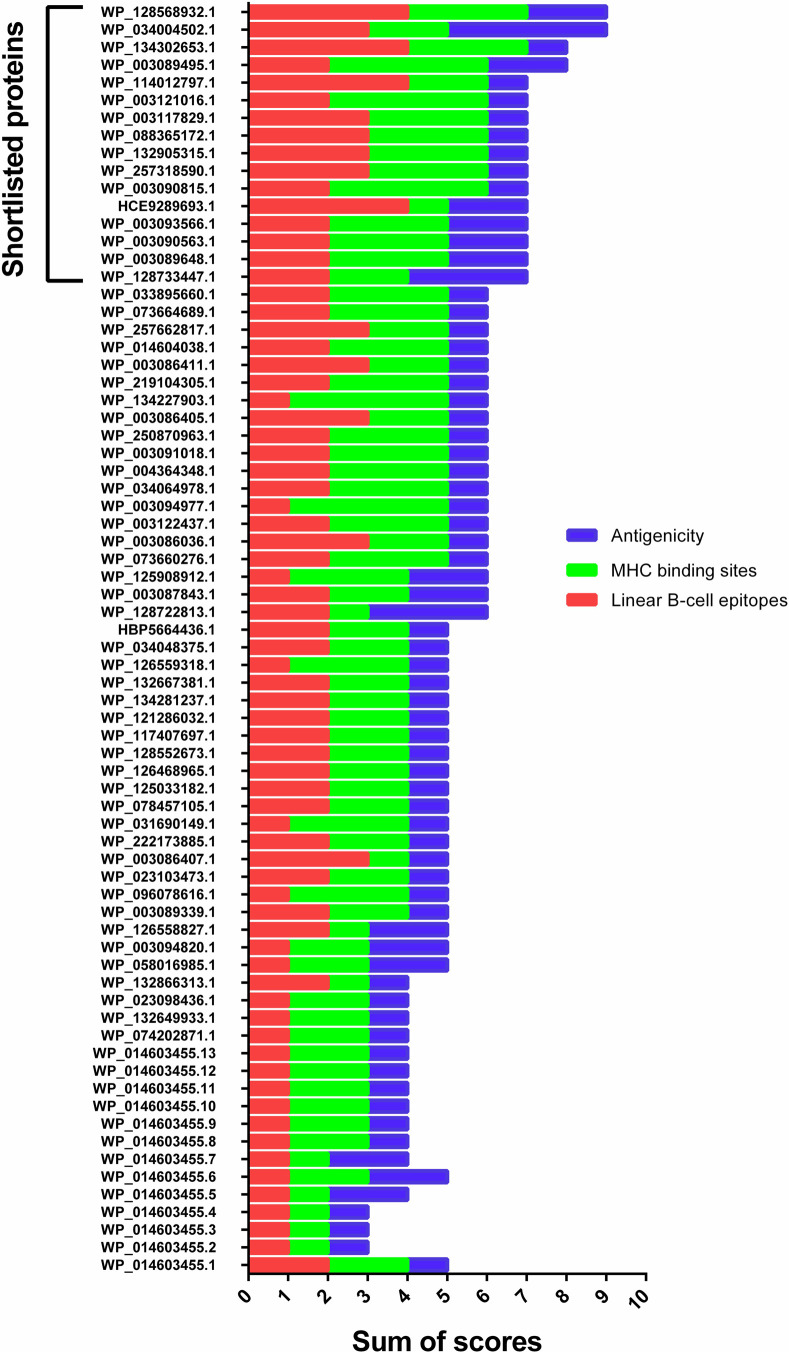
Comparative analysis of putative immunogenic targets against *P*. *aeruginosa* strain 24Pae112 based on the quartile scoring method. Sixteen proteins with a score ≥ 7 were selected, including Hypothetical proteins (WP_132548232.1 and WP_166796845.1), Type I fimbrial protein (WP_034004502.1), Type II secretion system secretin GspD (WP_061193930.1), Hcp family type VI secretion system effector (WP_110726056.1), TonB-dependent receptors (WP_023101627.1 and WP_125941151.1), Glycosyl hydrolase family 18 protein (WP_034004678.1), Type 4a pilus secretin PilQ (WP_132905315.1), Transporter (WP_024947839.1), OprD family porin (WP_003090815.1, WP_217385706.1, WP_019681707.1, and WP_243702750.1), Type III secretion system needle length determinant (WP_210733189.1), and Peptidoglycan-associated lipoprotein Pal (WP_058148283.1).

All selected proteins were assessed using the quartile scoring method. Finally, a total of 16 proteins (seven OMPs and nine secreted) with a contractual score ≥ 7 were considered shortlisted proteins and valuable for further investigations. The accession numbers for these proteins are as follows: Hypothetical proteins (WP_132548232.1 and WP_166796845.1), Type I fimbrial protein (WP_034004502.1), Type II secretion system secretin GspD (WP_061193930.1), Hcp family type VI secretion system effector (WP_110726056.1), TonB-dependent receptors (WP_023101627.1 and WP_125941151.1), Glycosyl hydrolase family 18 protein (WP_034004678.1), Type IVa pilus secretin PilQ (WP_132905315.1), Transporter (WP_024947839.1), OprD family porin (WP_003090815.1, WP_217385706.1, WP_019681707.1, and WP_243702750.1), Type III secretion system needle length determinant (WP_210733189.1), and Peptidoglycan-associated lipoprotein Pal (WP_058148283.1). See Fig 3.

### 3.5. Illustration of conformational B-cell epitopes and protein classification based on conserved domains

**Fig 4:**
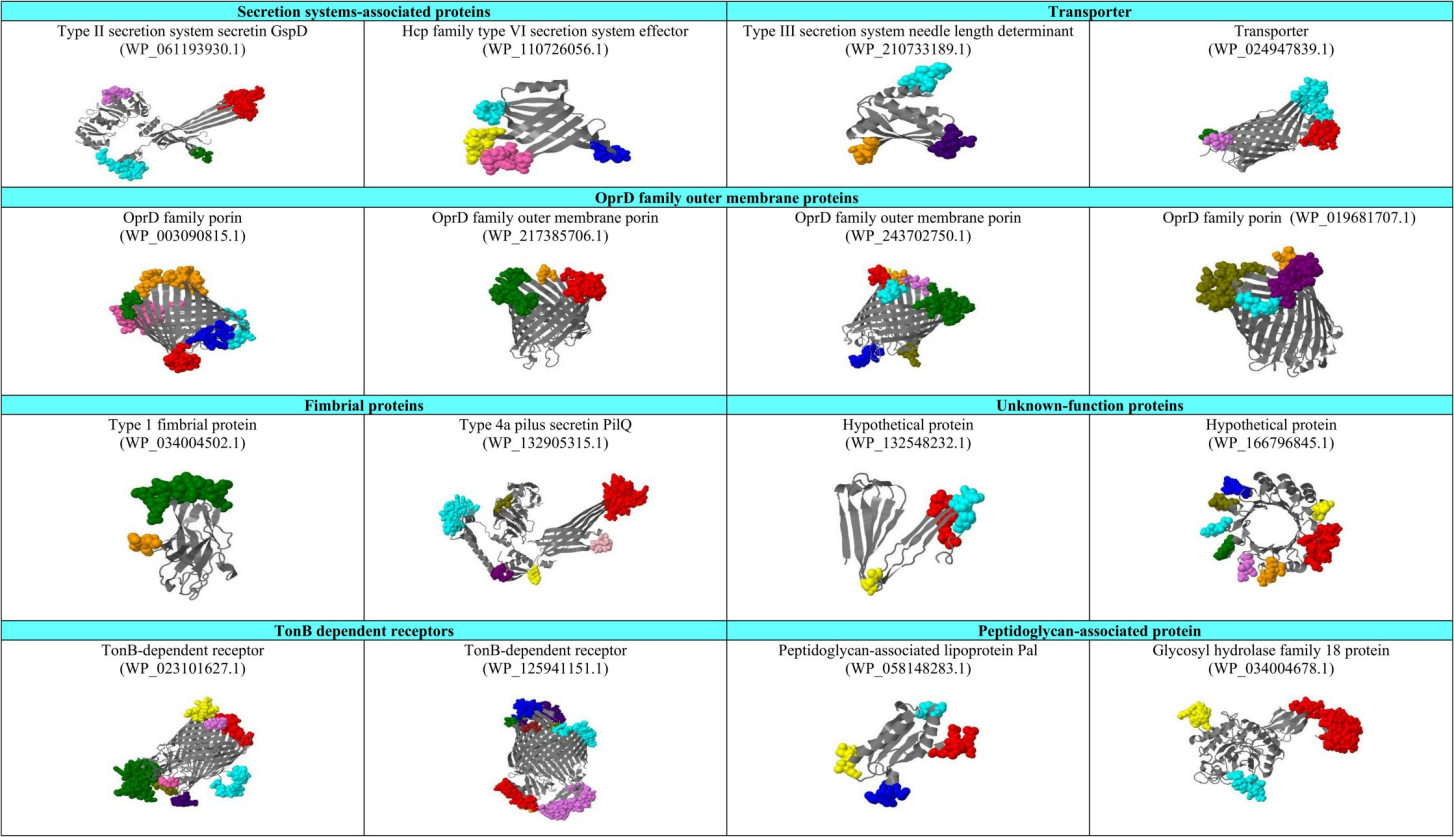
Surface-exposed conformational B-cell epitopes of putative immunogenic targets against *P*. *aeruginosa* strain 24Pae112 are illustrated in different colors. The tertiary structures of the proteins were predicted by the Robetta web tool, and the surface-exposed conformational B-cell epitopes were characterized by the 3D structure of proteins using Jmol software.

A quality assessment of the tertiary structures of proteins showed that the predicted structures are of acceptable quality. See S1 Fig. The 3D structure prediction of 16 shortlisted proteins and their conformational B-cell epitopes located on the surface of each protein is illustrated in Fig 4. Additionally, information about the predicted B-cell epitopes of seven OMPs is presented in Table 1. In the next step, proteins were classified into six different groups according to their conserved domains, including (I) Secretion system-associated proteins and transporters; (II) OprD family outer membrane proteins; (III) Fimbrial proteins; (IV) TonB-dependent receptors; (V) Peptidoglycan-associated proteins; and (VI) Unknown-function proteins (Hypothetical proteins).

**Table 1:** The sequence of linear and conformational B-cell epitopes of seven selected outer membrane proteins from *P*. *aeruginosa* strain 24Pae112.

No.	Protein name	Length (aa)	Linear B-cell epitopes (cut off ≥ 0.6)	Start	End	Number of epitopes	Epitope/amino acids ratio	Conformational B-cell epitopes	Score (cut off ≥ 0.8)	Color
1	WP_023101627.1 (TonB-dependent receptor)	964	PRMSGEAP	123	130	8	0.008	R596, D597, F600, I601, T602, V603, S604, R605, P606, G607, Y608, Y609, G610, S611, M612, M613, W614, F615, P616, D617, Q618, N619, G620, Q621, Y622, T623, D624, A625, T626, D627, P628, R629, L630, N631, N632, G633, I634, V635, T636, N637, N638, T639, N640, N641, P642, F643, E644, G645, I646, P647, F648, D649, E650, F651, G652, P653, A654, N655, V656, T657, V658, H659, P660, S661, R662, V663, T664, N665, V666, V667, R834, Q836, R837, T838, E839, N840, T841, N843	0.912	Green
			QKETYT	147	152			F508, E509, T510, D511, F512, G513, D514, F515, F563, P565, V566, E567, R568, L569, L571, F691, E692, L693, A694, P695, D696, T697	0.89	Yellow
			RNG	189	191			Q291, S294, A295, S296, S297, K298, T299, E300, N301, L302, S303, S304, V305, P306, H307, D308, D309, R310, G311, S312, F314, Q317	0.882	Cyan
			LV	269	270			R361, E365, S367, S368	0.87	Hot pink
			NGVAPQHRSASSKTENL	286	302			N329, E330, H331, L332, R396, I397, A398, D399, E400, H401, Y449, L450, P451, E452, N453, N454, P455, L456, V457	0.87	Red
			GSQAKSGSA	315	323			Y362, G363, R364, G426	0.827	Olive
			YRN	327	329			S746, L747, L748, A749, D750, G751, D752	0.825	Violet
			NQGNYFSGKKGQDRYRVYNRYGRE ESSVAKVYNAGEEVLNSSSE	342	385			P479, R480, S481, Q482, A483, Y484, R485, S486	0.812	Indigo
			RTGEI	413	417					
			PSDIFRFGTAGIYQYPLS	419	436					
			YLPENN	449	454					
			EAKSDMLTSVLAPR SQAYRSDRNWTRQD	467	494					
			EDIQPQKSVVTTLHDINANRTLRDA	526	550					
			NSKDNGISASPRREDRDMRFITVSRPGYYG SMMWFPDQNGQYTDATDP RLNNGIVTNNTNNPFEGI	581	646					
			FDEFGPANVTVHPSRVTNVVTGYNYSKKGSSRGG	648	681					
			LFETSQGTLQVE	712	723					
			DPGQM	774	778					
			TCDAAFAARLRAGANRYQRTENTPNC	819	844					
			GSYTNTQNP	851	859					
			ADKPWQVGATTPQ	893	905					
			PLAQSF	943	948					
2	WP_125941151.1 (TonB-dependent receptor)	953	AAKVQSLAAK	90	99	9	0.009	N774, A775, G776, D777, A778, S779, V780, D781, P782, N783, R784	0.918	Orange
			RAQQEG	116	121			D675, Y677, G678, C679, E680, K681, G682, V683, D684, C687, A750, A751, C752, R753, S754, G755, G756, L757, D758, P759, D760, S761, A762, C764, R765, L768	0.915	Red Blue
			GEDYGNTWQP	157	166					
			TAYGGSVNFT	195	204			V41, R42, G43, R44, G65, S66, G67, L68, Q69, A81, A82, P83, L84, R85, A86, A87, V88, P89, A90, A91, K92, V93, Q94, S95	0.895	Cyan
			QRQPVWANQRGFMDSYPD GNNVGYRLNPQSGRYLGD	288	323			D246, R721, W811, G812, A813, G814, D815, Y816, G817, Y865, A866, Q867, F868, Y901, L903, N904, E905, R906, A907, V909, F947, Y949, F951, G952, G953	0.881	Violet Indigo
			SCAGLGGLFGGNVAPVPGARGG YRCMTDQYYNNYWTLQT	325	363					
			QNNTRGPSF	396	404			R312, L313, N314, P315, Q316, S317, G318, R319, Y320, L321, G322, D323, A324, S325, C326, A327, G328, L329, G330, G331, L332, F333, G334, G335, N336, V337, A338, P339, P341, G342, A343, R344, G345, G346, Y347, I410, N411, A412, S413, S414, G415, D416, E428, D486, L489, G490, R491, K492, L493, G494, E495, R496, G498, P500, V501, Y502, D503, P504, D505, P506, A507, R508, L509, G510, R511, P512, L513, S514, E515, E516, E517, R519, R522	0.868	Olive brown Orange Red Blue Cyan
			PEEIGGKSSNN	426	436					
			DRVANT	452	457					
			YLGRKLGERDGYPVYDP	488	504					
			PARLGRPLSEEEWRSLRRNVTQ	506	527					
			IRPDSQYNDG	567	577					
			YNVSKASSSGG	579	589					
			FSGR	621	624			E8, A9, L10, T11, F12, D13, V14, A15, A16, G17, P18, Q45, A46, P47, A48, V49, R50, G51, P52, L53, S54, Q57, E60, Q61, R64, V454, A455, N456, T457	0.854	Violet Indigo
			QADANGYYPAQKDYYGCEKGVDGAC	663	687					
			KLLRDEAACRSGGLDPDSAQC	744	764			A274, T275, G276, D277, F278	0.851	Olive
			LARVERNAGDASVDPNRLN	768	786			P639, L640, T641, S642	0.822	Brown
			YQQSDDYPSENQRDSLDS	833	850			S39, D546, L547, P548, A549	0.817	Green
			VTNAAG	880	885					
			EDDSDGWPY	922	930					
3	WP_024947839.1 (transporter)	397	RQVEAQPQAPQPQRLVKSIQPPAQAR NDANAVAGTYGASLKDD	25	77	4	0.01	L116, L118, N119, G120, F121, L129, G130, N131, I132, G133, A174, G175, A176, G177, G178, S179, T180, S181, Q182, I183, T184, Q236, V237, P238, G239, N240, N241, N242	0.901	Cyan Green Violet
			GSTSQITEKSVTGD	178	191					
			PYGIKLKQVPGNNNLNVP	229	246					
			ESFDDINPQQGVKTG	285	299			V268, D269, P270	0.869	Red
			SKVKQDGQSWQTVSG	336	350			E208, S209, E210, S211, T212, P213	0.851	Cyan
								E284, D288, I290, N291, Q293, Q294, G295, V296, K297, T298, G299, K301, K339, Q340, D341, G342, Q343, S344, W345	0.831	Green
4	WP_003090815.1 (OprD family porin)	405	GTED	31	34	6	0.014	I212, P213, L214, Q215, A216, D217, Q218, I257, D258, A259, H260, F319, A320, K321, Y322, G323, V324, P325, G326, V360, Q361, S362, G363, P364, A365, K366, L368	0.872	Orange
			GKGRSGA	72	78			N1, Q3, E4, A5, A6, K7, G8, F9, V10, E11, D12, S13, S48, G49, F50, T51, Q52, G53, T54, I55, G56, I57, I107, S108, N109, E145, I146, E147, G148, V404, F405	0.868	Hotpink
			KQGDSGS	85	91			D232, S233, D234, A236, D237, Q238, N239, N241, G242, N243, R244, D272	0.849	Red
			RKSAEGRDS	161	169			F184, T185, D186, H187	0.832	Green
			DFADQNFNGN	234	243			K340, T341, A342, E343, T344, S345, N346, S380, A381, D382, D385	0.811	Blue
			TAET	341	344			L69, D70, G71, G72, K73, Q86, G87, D88, S89, G90, S91	0.81	Cyan
			DARDSYFS	382	389					
5	WP_217385706.1 (OprD family outer membrane porin)	400	DGSSANPQGASK	23	34	3	0.007	A1, F2, L3, E4, D5, G6, S48, G49, Y50, T51, E52, G53, A54, L55, G56, F57, M104, F106, S107, Q108, E144, I145, A146, G147	0.898	Red
			ADSN	74	77			R224, Q225, L226, G227, A228, G229, K230, A263, Q264, G265, G266, H267, F315, L317, R318, S319, V320, G321, V322, P323, G324, V358, Q359, S360, G361, R362, F363, K364, D365	0.883	Green Red Green
			HDPRR	86	90					
			ARDSSDAQDIRLHCKNKRYACD	160	181					
			AGAARAG	243	249					
			LAN	339	341			V196, N197, D198, G199	0.839	Orange
			ERSA	380	383					
6	WP_243702750.1 (OprD family outer membrane porin)	411	ADAGRG	29	34	8	0.019	L307, A308, P309, F310, G311, L312, P313, G314	0.907	Red
			GHA	75	77			T2, E3, E4, A5, K6, A7, P8, D9, Y10, L11, Y51, T52, P53, G54, R55, V56, G57, F58, R104, L105, G106, E107, D143, L144, D145, R146, G409, S410, L411	0.905	Green
			SGDNA	87	91			V254, G255, A256, Q257	0.894	Yellow
			EQASSSGHGDFDGYGAS	159	175			A190, S191, D192, N193, R218	0.879	Violet
			QGRSRAGA	233	240			A219, A220, F221, Y252	0.87	Orange
			GSHAPAGGAYNPLGADGRYRPLQGSG	329	354			A334, N339, P340, L341, G342, A343, D344, G345, R346, Y347	0.865	Blue
			AQAG	390	393			F368, A369, S370, G371, P372, L373, K374, L376	0.837	Cyan
								N205, Q233, G234, R235, S236	0.819	Olive
7	WP_019681707.1 (OprD family porin)	447	TPSRRD	50	55	4	0.008	R247, L248, S249, S250, Q251, L252, T253, L254, G287, G288, G289, Q290, L343, A344, F345, L346, A347, A348, P349, D350, W351	0.914	Violet
			GGSGG	94	98					
			GR	108	109			V24, P25, P26, G27, F28, V29, E30, G31, S32, S69, G70, Y71, T72, D73, T74, P75, I76, G77, V78, L123, R124, G125, L126, D127, Y162, H163, F164, L446, L447	0.865	Green
			LRNQSGHS	179	186					
			GNGTKGGIAADR	193	204					
			DQGRSQLG	265	272			A218, P219, G220, G221, S222, Q244, W246	0.839	Cyan
			TRVDPDSAG	365	373			F398, P399, A400, G401, P402, A403, K404, G405, L406	0.807	Orange
			YTAPGNTRGNS	421	431					

### 3.6. Protein-protein interaction networks

**Fig 5:**
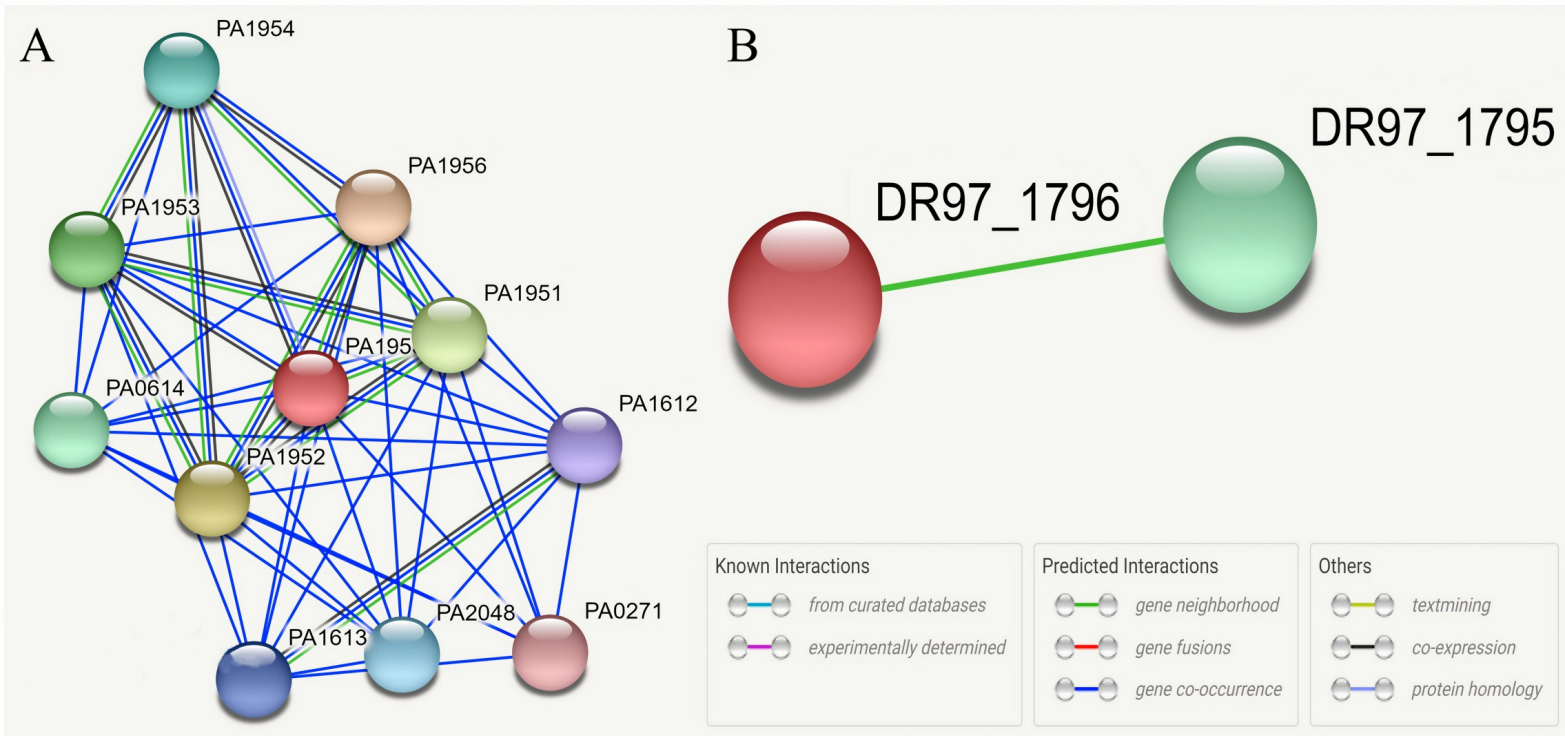
Protein-protein STRING interaction networks of two Hypothetical proteins (e.g. WP_132548232.1 and WP_166796845.1) with other proteins of P. aeruginosa strain 24Pae112. According to obtained result, WP_132548232.1 has co-occurrence and co-expression with Peptidase C39 domain-containing protein (PA1953) and co-occurrence with ABM domain-containing protein (PA0271).

According to the obtained results from the STRING database, the Hypothetical protein WP_132548232.1 had co-occurrence and co-expression with peptidase C39 domain-containing protein (PA1953) and co-occurrence with ABM domain-containing protein (PA0271). On the other hand, the Hypothetical protein WP_166796845.1 is neighboring the UvrD/REP helicase N-terminal domain protein (DR97_1795). The full results of protein-protein interaction networks are presented in Fig 5.

### 3.7. Designing multiple-epitope-based vaccines

**Fig 6:**
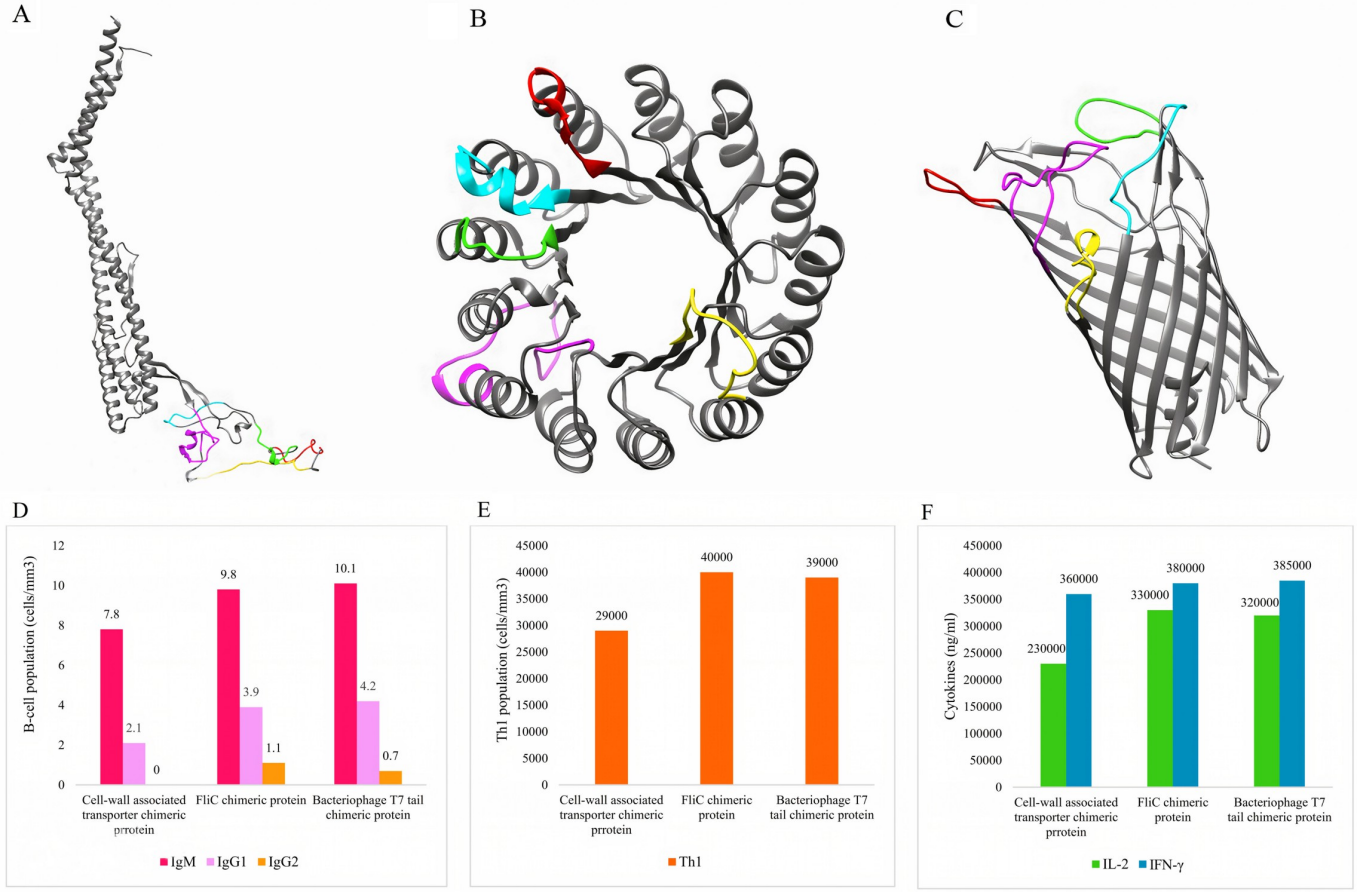
The 3D structures of the multi-epitope vaccines were predicted using the Robetta web tool and validated by the ProSA web server. The linear B-cell epitopes are colored using Jmol software (A-C). In addition, immune simulation of designed vaccines has shown in the Figure (D-E). **A)** The structure and epitopes of chimeric FliC protein, **B)** The bacteriophage T7 tail, and **C)** The cell wall-associated chimeric proteins. **D)** Bacteriophage T7 tail chimeric protein and FliC chimeric protein induced higher levels of IgM, IgG1, and IgG2. **E)** FliC chimeric protein and bacteriophage T7 tail chimeric protein showed higher levels of Th1 cell population production. **F)** A higher level of IL-2 and IFN-γ was induced by the bacteriophage T7 tail and FliC chimeric proteins.

Five conserved and highly antigenic B-cell epitopes, including PEEIGGKSSNN, YNVSKASSSGG, QADANGYYPAQKDYYGCEKGVDGAC (obtained from TonB-dependent receptor), GSTSQITEKSVTGD (extracted from Transporter), and YTAPG NTRGNS (obtained from OprD family porin), with rigid (EAAAK) and flexible (GPGPG) linkers, were assessed through different arrangements, and the most antigenic arrangement was selected to generate the chimeric FliC protein. To generate chimeric proteins from the bacteriophage T7 tail (WP_166796845.1) and the cell-wall-associated transporter (WP_024947839.1), the mentioned epitopes were located in the disordered regions of these platforms. The detailed data are provided in the S2 Table. The 3D structure of the multi-epitope proteins is demonstrated in Fig 6.

### 3.8. Molecular docking and *in silico* immunization

**Fig 7:**
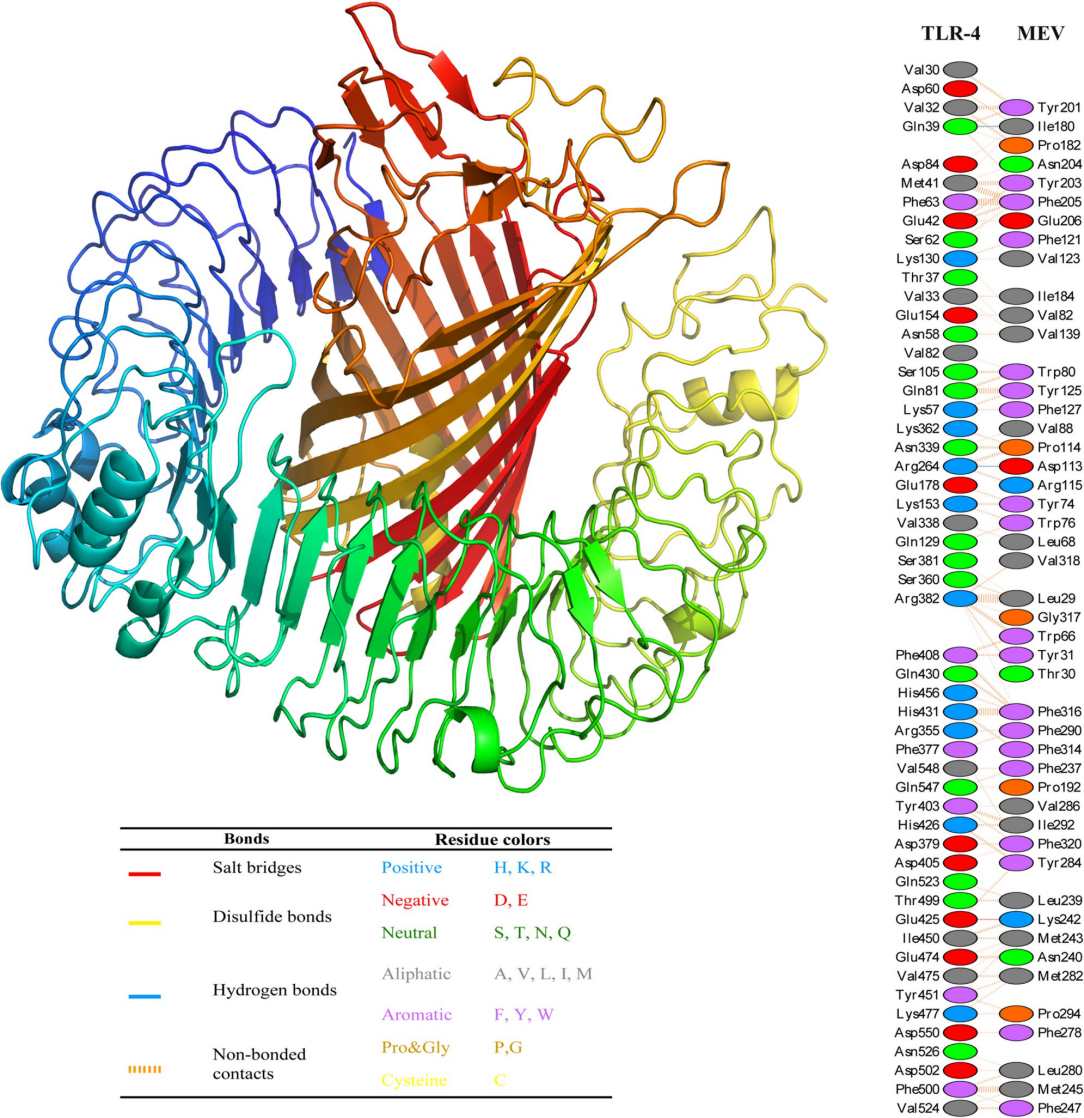
Details of interactions between the chimeric cell-wall-associated transporter and TLR4. The 3D structure prediction of the chimeric cell-wall-associated transporter-TLR4 complex was illustrated using the UCSF Chimera software. The paired residue’s interactions between cell-wall-associated transporter and TLR4 have shown with different colors (Table is near here.) in the Figure.

The pyDockWEB results demonstrated that the chimeric cell-wall-associated transporter as a multi-epitope vaccine had the strongest interactions with TLR1 (-67.516 kcal/mol), TLR2 (-52.280 kcal/mol) and TLR4 (-69.791 kcal/mol). The full results of this step are presented in Table 2. In addition, the C-ImmSim data revealed that FliC and bacteriophage T7 tail chimeric proteins induced higher levels of immunoglobulins (*e*.*g*. IgM and IgG1), Th1 cell population, and cytokines (*e*.*g*. IFN-γ and IL-2) in comparison to the cell-wall-associated transporter chimeric protein. See Fig 6. In addition, the details of interactions between cell-wall-associated transporter and TLR4 are presented in Fig 7.

**Table 2:** The thermodynamic interactions of three proposed multi-epitope-based vaccines against *P*. *aeruginosa* with TLR1, 2, and 4.

Interactions of multi-epitope-based vaccines and TLRs	Total (kcal/mol)	VdW (kcal/mol)	Desolvation (kcal/mol)	Electrostatics (kcal/mol)
FliC -TLR1	-29.006	11.341	-4.547	-25.593
FliC -TLR2	-31.891	30.684	1.918	-36.878
FliC -TLR4	-35.018	23.241	-17.548	-19.794
Bacteriophage T7 tail protein-TLR1	-26.392	-6.142	-0.330	-25.447
Bacteriophage T7 tail protein-TLR2	-23.688	0.488	-11.324	-12.413
Bacteriophage T7 tail protein-TLR4	-32.649	30.339	-21.364	-14.320
Cell-wall associated transporter-TLR1	-67.516	17.263	-46.745	-22.497
Cell-wall associated transporter-TLR2	-52.280	12.465	-40.360	-13.166
Cell-wall associated transporter-TLR4	-69.791	138.816	-69.328	-14.344

*VdW: Van-der-Waals

### 3.9. Molecular dynamics simulation of the chimeric cell-wall associated transporter in complex with the TLR4

**Fig 8:**
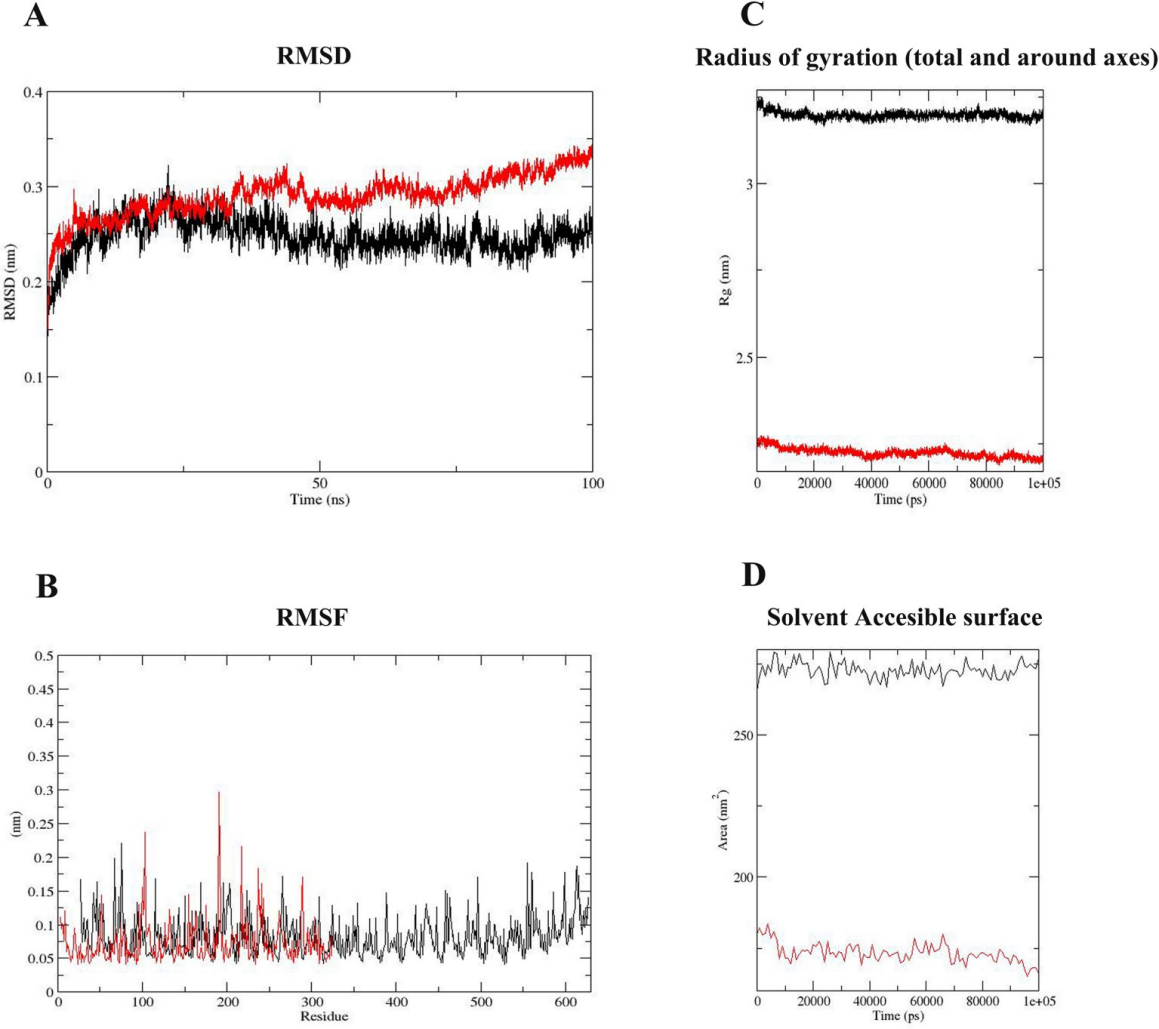
Molecular dynamics (MD) simulation results. **A)** According to the RMSD plot, TLR4 and cell-wall- associated transporter were in the range of 0.2 to 0.27 and 0.2 to 0.3, respectively. This result indicates that both proteins reach stability at time 10 and remain physically stable after that. **B)** The obtained result from the RMSF plot demonstrated that no unusual fluctuation was observed in protein structures. **C)** According to the Rg result, the structures of cell-wall-associated transporter and TLR4 were stable. **D)** The SASA of cell-wall-associated transporter and TLR4 was in the range of 168 to 18 nm^2^ and 270 to 277 nm^2^, respectively. In all Figures, TLR4 and complex (including TLR4 and chimeric cell-wall-associated transporter) are presented in red and block colors, respectively.

The global structural stability of the cell-wall associated transporter-TLR4 complex was evaluated using a Root Mean Square Deviation (RMSD) plot. This plot shows how much the protein conformation has changed during MD simulation from the initial structure. The RMSD of TLR4 and cell-wall-associated transporter was in the range of 0.2 to 0.27 and 0.2 to 0.3, respectively. Both proteins reach stability at time 10 and remain stable after that (Fig 8A). The Root Mean Square Fluctuation (RMSF) indicates the fluctuation of protein residues over time from a reference position during simulation. According to the RMSF plot, no unusual fluctuation was observed in protein structures (Fig 8B). The compactness of the cell-wall-associated transporter-TLR4 complex was evaluated using the Radius of gyration (Rg) plot. The Rg of TLR4 was in the range of 3.19 to 3.24. While the Rg of cell-wall-associated transporter was in the range of 2.25 to 2.2. According to the result, the structures of both proteins were stable (Fig 8C). Furthermore, the Solvent Accessible Surface Area (SASA) for both proteins was calculated during the simulation. SASA indicates the accessible surface of the protein solvent or a part of a protein that is exposed to the solvent. The SASA of cell-wall-associated transporter was in the range of 168 to 18 nm^2^. While the SASA of TLR4 was in the range of 270 to 277 nm^2^ (Fig 8D).

## 4. Discussion

Nowadays, one of the most important challenges for physicians is the effective treatment of infections caused by Gram-negative pathogens due to the increasing antibiotic resistance in healthcare. Meanwhile, *P*. *aeruginosa* occupies a leading role among infections caused by Gram-negative rods, particularly in critically ill and immunocompromised patients. Due to antimicrobial resistance, there are severely restricted treatment options for *P*. *aeruginosa* infection, which has become a critical and fatal problem [47, 48]. To date, many studies have been conducted in pre-clinical and clinical trial stages to introduce an effective vaccine against this bacterium. Despite all efforts, there are still no *P*. *aeruginosa* FDA-approved vaccines for human administration, and recent promising candidates have failed in the final steps of clinical trials [49].

In this context, early vaccines against this bacterium targeted the lipopolysaccharide (O-antigen) and could generate satisfactory immunity. However, this protection was LPS serotype-specific, and with more than 30 subtypes of O-antigens, this strategy has met significant challenges [50]. In addition, vaccination with the type III secretion system protein PopB encapsulated into PLGA (polylactic-co-glycolic acid) nanoparticles revealed protection against the lethal *P*. *aeruginosa* challenge and induced a Th17 response in the mouse model [50]. However, vaccine-induced Th17 responses may be harmful in the situation of cystic fibrosis (CF), because Th17-characterized lung inflammation has been observed in CF patients [51]. Moreover, it was reported that the killed but metabolically active (KBMA) attenuation *P*. *aeruginosa*-based vaccine induces preventative immunity responses against pulmonary infections [11]. It seems that this type of vaccine has some disadvantages, including the risk of reversion to a virulent strain and safety concerns for immunocompromised people [52]. In addition, the high amount of LPS and its toxicity are other points that should be considered [53].

So far, different *in silico* studies have been conducted to introduce immunogenic targets against this bacterium. For example, Beg *et al*. designed multi-epitope-based vaccines against functional amyloids of *P*. *aeruginosa* (Fap) using different immunoinformatic and structural bioinformatic approaches [54]. In addition, the computational study conducted by Dey *et al*. mainly focused on *P*. *aeruginosa’s* major membrane proteins, including OprF and OprI, to design peptide-based vaccine constructs [55]. In addition, Elhag and colleagues predicted an epitope-based vaccine against this bacterium using fructose bisphosphate aldolase (FBA) protein through immunoinformatics tools [56]. Although the results of these studies were valuable in finding a promising vaccine against this bacterium, their major limitation was their focus on just one or two bacterial proteins. Meanwhile, the study by Solanki *et al*. was more comprehensive, as they used a comparative subtractive proteomic analysis among 1,191 *P*. *aeruginosa* proteomes and finally left a total of twenty unique and non-redundant proteomes. However, they presented a chimeric vaccine with PADRE (pan-HLA-DR epitopes) as an adjuvant [57] which is completely different from our presented platforms.

In this comprehensive study, it was considered the total proteins of *P*. *aeruginosa* 24Pae112 and evaluated them from all aspects of immunoinformatics to find novel and putative immunogenic targets. In this regard, different bioinformatics tools and algorithms were used to justify the obtained results. In the case of antigenicity and allergenicity, for example, we used Vaxigen/ANTIGENpro and AlgPred/AllergenFP, respectively. However, we considered only one of them in the study since VaxiJen is the first server for alignment-independent prediction of protective antigens and has been used for antigenicity prediction in many articles on reverse vaccinology [58–60]. In the case of allergenicity, it was found that the results from both databases largely overlapped (only in seven cases were the results different) and we considered the results obtained from AlgPred. Moreover, in the present study, the quartile ranking method was used to select the shortlisted proteins. It should be mentioned that quartile analysis can be a valuable global approach in reverse vaccinology because when selecting the best targets, different criteria such as antigenicity, allergenicity, *etc*. can be taken into account at the same time [61].

The authors of this article believe that using multiple tools for analysis will not always yield the desired results. For example, in subcellular localization, the simultaneous use of several tools to determine the location of proteins in bacteria will be confusing due to different prediction algorithms. Furthermore, we attempted to choose experimental-based tools. However, the accuracy rate of computational tools can be considered as a limitation.

The results of this study are significant, as all 16 shortlisted proteins can be obtained as subunit vaccines for further studies in animal models. These proteins are surface-exposed, have a molecular weight of < 110 kDa, and are capable of inducing both humoral and cellular immunity.

The first group of shortlisted putative vaccine candidates belong to secretion systems and transports, including (I) Type II secretion system secretin GspD (WP_061193930.1), (II) Hcp family type VI secretion system effector (WP_110726056.1), (III) Type III secretion system needle length determinant (WP_210733189.1), and (IV) Transporter (WP_024947839.1). The transport of proteins from the cytoplasm to other parts of the cell or the environment, a process known as secretion, is one of the most important prokaryotic cell functions. Prokaryotes have evolved numerous ways to transport protein cargo between locations, most of which involve the assistance of specialized protein secretion systems. Proteins taking part in secretion systems are essential for bacterial growth and are involved in several processes [62]. Proteins from this group have already been introduced as vaccine candidates against this bacterium. For example, it was reported that immunization of mice with surface-expressed PcrV, the needle-tip protein component of T3SS, resulted in a reduction in cytotoxicity and inflammation and enabled neutrophil internalization of *P*. *aeruginosa* [63, 64]. Therefore, it can be concluded that secretion system-associated proteins can play an important role in immunogenicity in addition to pathogenicity in this bacterium.

In the second group, four proteins, including WP_003090815, WP_217385706.1, WP_243702750.1, and WP_019681707.1, belong to the OprD family of outer membrane proteins. This family was identified for the first time during outer membrane investigations of carbapenem-resistant *Acinetobacter baumannii* isolates [65]. The OprD is an orthologous protein and the prototype of a specific channel superfamily notable for its 19 members in *P*. *aeruginosa*. These proteins demonstrated 46 to 57% similarity and play an important role in amino acid or organic acid uptake. The OprD has been extensively studied based on its structure, function, and involvement in the carbapenem resistance of *P*. *aeruginosa*. This protein, with an 18-strand barrel structure, forms a very narrow channel with two structural features that may contribute to its channel specificity [66, 67].

In the third group, we introduced fimbrial proteins including, (I) Type I fimbrial protein (WP_034004502.1) and (II) Type IVa pilus secretin PilQ (WP_132905315.1) as a putative vaccine candidate against *P*. *aeruginosa*. This protein group was previously introduced as vaccine candidates against other Gram-negative bacteria such as *Escherichia coli* [68], *Klebsiella pneumoniae* [69], and *Salmonella enterica* serovar Typhi [69]. Moreover, a study conducted by Gholami *et al*. reported the potential of integrated PilQ/PilA (QA) antigens as a promising vaccine candidate against *P*. *aeruginosa*. Those study revealed that the chimeric protein PilQ and the disulfide-turn region of PilA trigger the production of specific antibodies in the BALB/c mouse model [70].

In the fourth group, TonB-dependent receptors (WP_023101627.1 and WP_125941151.1) were presented as putative vaccine candidates against *P*. *aeruginosa*. TonB-dependent receptors are a family of beta-barrel proteins named for their localization in the outer membrane of Gram-negative bacteria. These complexes recognize various signals from outside of the bacterial cells and transduce them into the cytoplasm, resulting in transcriptional activation of target genes [71]. This well-known protein has been previously introduced as a putative vaccine candidate against several Gram-negative bacteria, such as *A*. *baumannii* [72], *N*. *gonorrhoeae* [73], and *K*. *pneumoniae* [74].

In the fifth group, peptidoglycan-associated proteins, including (I) Peptidoglycan-associated lipoprotein Pal (WP_058148283.1) and (II) Glycosyl hydrolase family 18 protein (WP_034004678.1), are among the shortlisted immunogenic targets against *P*. *aeruginosa*. In Gram-negative bacteria, Pal is related to the integrity of the cellular envelope and interacts powerfully with the peptidoglycan layer [75]. Consistent with our results, it was recently reported that the peptidoglycan-associated lipoprotein Pal is a critical virulence determinant of *Burkholderia mallei* and could be considered an effective target for developing a vaccine against glander [76].

Finally, the Hypothetical protein WP_132548232.1 has co-occurrence and co-expression with peptidase C39 domain-containing proteins and co-occurrence with ABM domain-containing proteins. The peptidase C39 domain-containing protein defined by this cysteine peptidase domain belongs to the peptidase family C39 (clan CA). It is found in a wide range of ABC transporters, which are maturation proteases for peptide bacteriocins, with the proteolytic domain located in the N-terminal region of the protein [77]. On the other hand, the Hypothetical protein WP_166796845.1 is neighboring the UvrD/REP helicase N-terminal domain protein. UvrD helicase is a multi-domain DNA helicase that has a molecular weight of 82 kDa [78].

A multi-epitope vaccine composed of a series of peptides is an ideal approach for the prevention and treatment of bacterial or viral infections [79]. Introducing cell-wall-associated transporter and bacteriophage T7 tail chimeric proteins as multi-epitope vaccines against *P*. *aeruginosa* and suitable scaffolds for multi-epitope vaccine design and development was the significant novelty of the present study. These new platforms for vaccine design can be used not only against *P*. *aeruginosa* but also against other bacteria. The disordered regions of these proteins are proper for the implantation of conserved and antigenic epitopes from different pathogens to induce a satisfactory immune response. Cell-wall-associated transporter had the strongest interactions with TLR 1, 2, and 4. TLRs are the major mediators of inflammatory pathways, playing important roles in mediating immune responses to a variety of pathogen-derived ligands and linking adaptive immunity to innate immunity [80]. On the other hand, the bacteriophage T7 tail chimeric protein had the strongest ability to stimulate the immune response. This result showed the better performance of these two platforms compared to the FliC, which is a known platform for multi-epitope vaccine design [81].

## 5. Conclusion

This comprehensive study evaluated the entire genome sequence of *P*. *aeruginosa* strain 24Pae112 to find novel and putative immunogenic targets. The results of this study are significant, as all 16 proteins presented are valuable for further investigation in animal models. These proteins were classified into six different groups according to their conserved domains, including (I) Secretion system-associated proteins and transporters, (II) OprD family outer membrane proteins, (III) Fimbrial proteins, (IV) TonB dependent receptors, (V) Peptidoglycan-associated proteins, and (VI) Unknown-function proteins (Hypothetical proteins). On the other hand, three designed multi-epitope vaccines showed promising results. Finally, it is worth noting that two proteins (*e*.*g*. cell-wall-associated transporter and bacteriophage T7 tail chimeric protein) as new platforms for vaccine design and development can be used not only against *P*. *aeruginosa* but also against other superbugs. We hope these two platforms will receive more investigation in future studies.

## Supporting information

S1 Fig Quality assessment of tertiary structures of 16 shortlisted proteins using ProSA web server and Ramachandran plots. (PDF)

S1 Table The molecular weight, functional class, conserved domain, and prevalence prediction of 72 selected proteins against *P*. *aeruginosa* 24Pae112. (DOCX)

S2 Table Evaluation of linear B-cell epitopes and design of different multi-epitope vaccines against *P*. *aeruginosa* 24Pae112. (DOCX)
