# Supplementary material for: Defeating a superbug: A breakthrough in vaccine design against multidrug-resistant Pseudomonas aeruginosa using reverse vaccinology
Source: PLoS One. 2023 Aug 3;18(8):e0289609. doi: 10.1371/journal.pone.0289609 (PMC10399887; doi:10.1371/journal.pone.0289609)
Supplement: S1 Fig — (PDF) [file pone.0289609.s001.pdf]

S2 Fig. Quality assessment of tertiary structures of 16 shortlisted proteins using ProSA web server and Ramachandran plots.

Secretion systems-associated proteins

Type II secretion system secretin GspD
(WP_061193930.1)

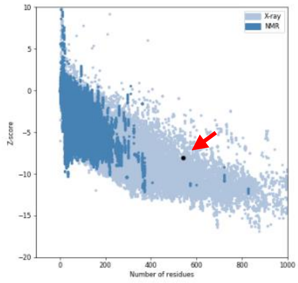

Z-score: -8.08

Hcp family type VI secretion system effector
(WP_110726056.1)

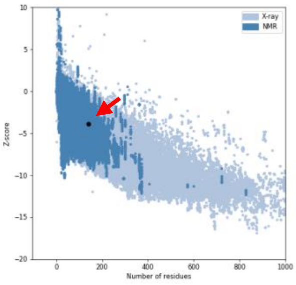

Z-score: -3.86

Transporter

Type III secretion system needle length determinant
(WP_210733189.1)

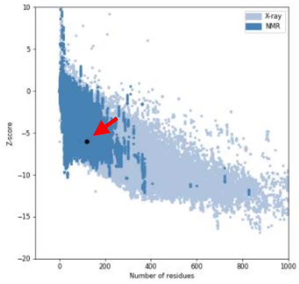

Z-score: -5.99

Transporter
(WP_024947839.1)

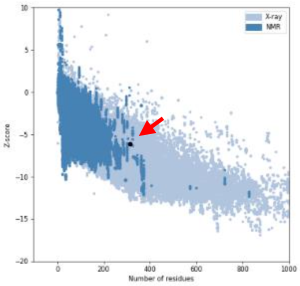

Z-score: -6.13

OprD family outer membrane proteins

OprD family porin
(WP_003090815.1)

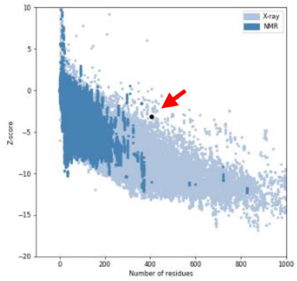

Z-score: -3.17

OprD family outer membrane porin
(WP_217385706.1)

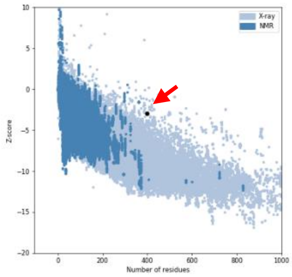

Z-score: -2.91

OprD family outer membrane porin
(WP_243702750.1)

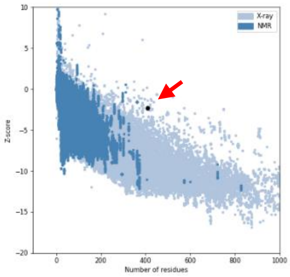

Z-score: -2.26

OprD family porin
(WP_019681707.1)

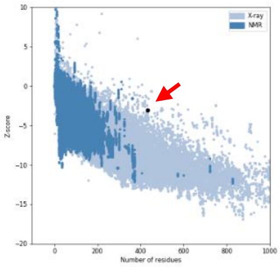

Z-score: -3

Fimbrial proteins

Type 1 fimbrial protein
(WP_034004502.1)

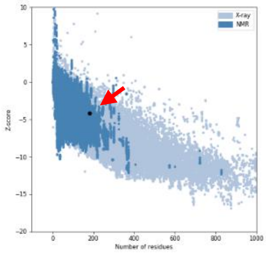

Z-score: -4.18

Type 4a pilus secretin PilQ
(WP_132905315.1)

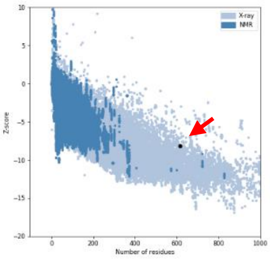

Z-score: -8.12

Peptidoglycan-associated protein

Peptidoglycan-associated lipoprotein Pal
(WP_058148283.1)

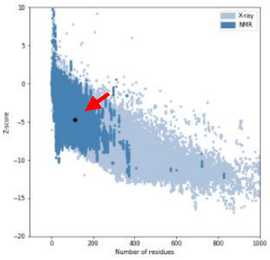

Z-score: -4.68

Glycosyl hydrolase family 18 protein
(WP_034004678.1)

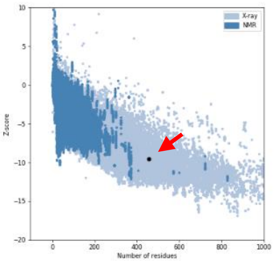

Z-score: -9.52

TonB dependent receptors

TonB-dependent receptor
(WP_023101627.1)

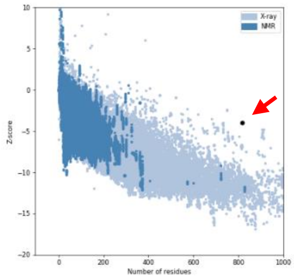

Z-score: -3.99

TonB-dependent receptor
(WP_125941151.1)

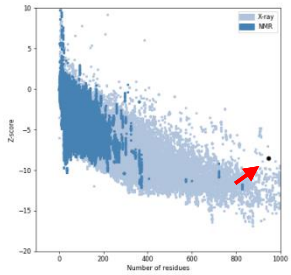

Z-score: -8.47

Unknown-function proteins

Hypothetical protein
(WP_132548232.1)

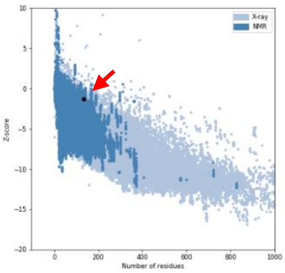

Z-score: -1.25

Hypothetical protein
(WP_166796845.1)

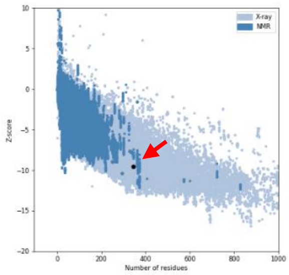

Z-score: -9.53

Secretion systems-associated proteins

Type II secretion system secretin GspD
(WP_061193930.1)

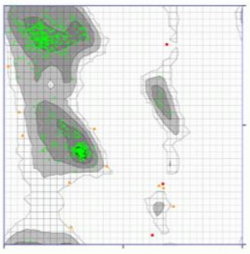

Highly Preferred zones:97.18%
Preferred zones:2.16%
Questionable zones:0.64%

Hcp family type VI secretion system effector
(WP_110726056.1)

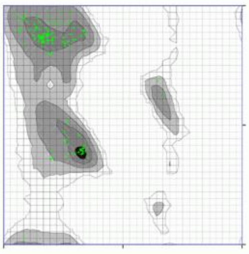

Highly Preferred zones:100 %
Preferred zones: 0%
Questionable zones:0%

Transporter

Type III secretion system needle length determinant
(WP_210733189.1)

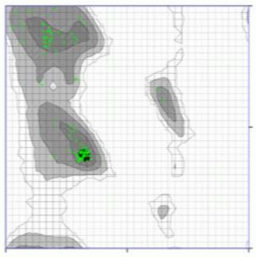

Highly Preferred zones:100%
Preferred zones:0%
Questionable zones:0%

Transporter
(WP_024947839.1)

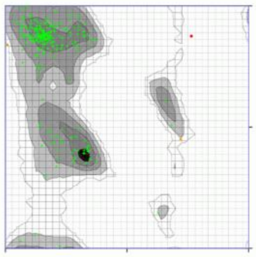

Highly Preferred zones:98.83%
Preferred zones:0.77%
Questionable zones:0.38%

OprD family outer membrane proteins

OprD family porin
(WP_003090815.1)

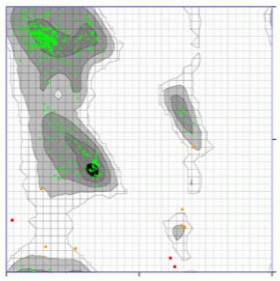

Highly Preferred zones:97.16%
Preferred zones:1.98%
Questionable zones:0.85%

OprD family outer membrane porin
(WP_217385706.1)

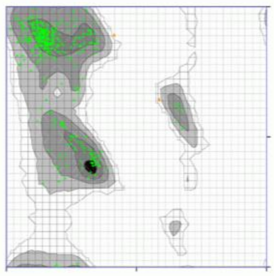

Highly Preferred zones:99.42%
Preferred zones:0.57%
Questionable zones:0%

OprD family outer membrane porin
(WP_243702750.1)

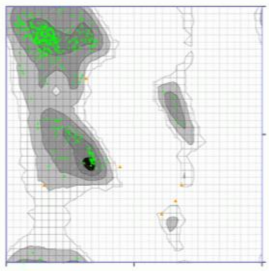

Highly Preferred zones:98.25%
Preferred zones: 1.74%
Questionable zones:0%

OprD family porin
(WP_019681707.1)

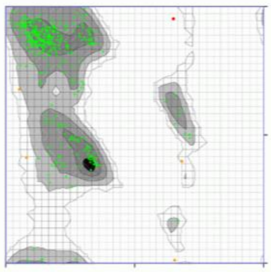

Highly Preferred zones:98.58%
Preferred zones:1.13%
Questionable zones:0.28%

Fimbrial proteins

Type 1 fimbrial protein
(WP_034004502.1)

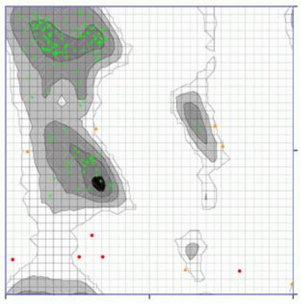

Highly Preferred zones:92.46%
Preferred zones:4.11%
Questionable zones:3.42%

Type 4a pilus secretin PilQ
(WP_132905315.1)

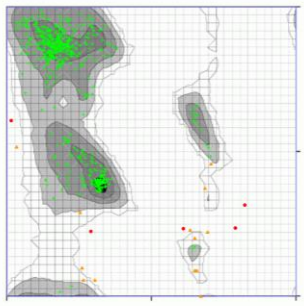

Highly Preferred zones:96.64%
Preferred zones:2.42%
Questionable zones:0.93%

Peptidoglycan-associated protein

Peptidoglycan-associated lipoprotein Pal
(WP_058148283.1)

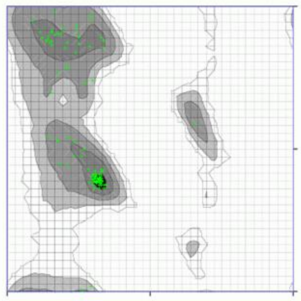

Highly Preferred zones: 100%
Preferred zones:0%
Questionable zones:0%

Glycosyl hydrolase family 18 protein
(WP_034004678.1)

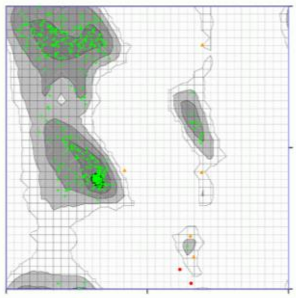

Highly Preferred zones:98.19%
Preferred zones:1.28%
Questionable zones:0.51%

TonB dependent receptors

TonB-dependent receptor
(WP_023101627.1)

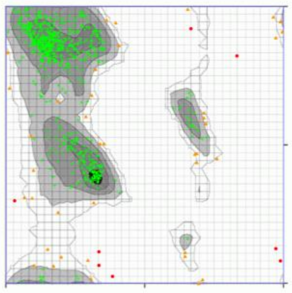

Highly Preferred zones:93.3%
Preferred zones:5.55%
Questionable zones:1.14%

TonB-dependent receptor
(WP_125941151.1)

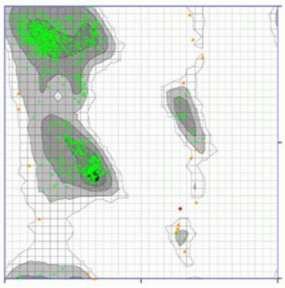

Highly Preferred zones:97.75%
Preferred zones:2.12%
Questionable zones:0.12%

Unknown-function proteins

Hypothetical protein
(WP_132548232.1)

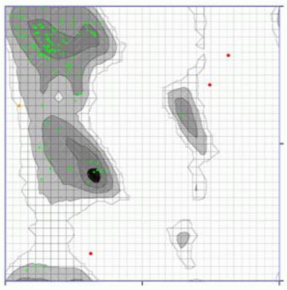

Highly Preferred zones:96.46%
Preferred zones:0.88%
Questionable zones:2.65%

Hypothetical protein
(WP_166796845.1)

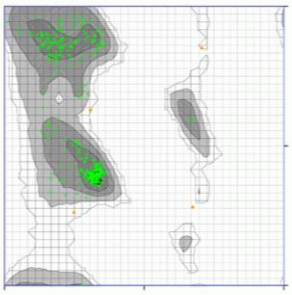

Highly Preferred zones:98.76%
Preferred zones:1.23%
Questionable zones:0%
