# Supplementary material for: Defeating a superbug: A breakthrough in vaccine design against multidrug-resistant Pseudomonas aeruginosa using reverse vaccinology
Source: PLoS One. 2023 Aug 3;18(8):e0289609. doi: 10.1371/journal.pone.0289609 (PMC10399887; doi:10.1371/journal.pone.0289609)
Supplement: S1 Table — (DOCX) [file pone.0289609.s002.docx]

**S1 Table.** The molecular weight, functional class, conserved domains, and prevalence prediction of 72 selected proteins against *P. aeruginosa* 24Pae112.

| **No.** | **Protein** | **Subcellular localization**  **(Psortb)** | **Transmembrane**  **(TMHMM - 2.0)** | **Antigenicity**  **(Vaxigen)** | **Antigenicity**  **(ANTIGENpro)** | **Hybrid score** | **Allergenicity**  **(AlgPred)** | **Allergenicity**  **(AllergenFP)** | **VICMpred** | **EggNOG** | **CD-search** | **Prevalence** | **MW**  **(kDa)** |
| --- | --- | --- | --- | --- | --- | --- | --- | --- | --- | --- | --- | --- | --- |
| 1 | WP_014603455.1 | Outer Membrane (10.00) | Outside | 0.7105  (Probable ANTIGEN) | 0.517971 | 0.05 | Non-Allergen | PROBABLE NON-ALLERGEN | Virulence factors  (1.8583441) | Intracellular trafficking, secretion, and vesicular transport  hemolysin activation secretion protein | FhaC  Hemolysin activation/secretion protein | 100% | 63.27 |
| 2 | WP_162904301.1 | Outer  Membrane (9.99) | Outside | 0.8122  (Probable ANTIGEN) | ND | 0.19 | Non-Allergen | PROBABLE NON-ALLERGEN | Information and Storage  (-2.0966323) | Intracellular trafficking, secretion, and vesicular transport  Large exoproteins involved in heme utilization or adhesion | ND | 76.39% | 365.26 |
| 3 | WP_132905315.1 | Outer  Membrane (10.00) | Outside | 0.6621  (Probable ANTIGEN) | 0.881465 | 0.14 | Non-Allergen | PROBABLE NON-ALLERGEN | Cellular process  (-5.2273836) | Intracellular trafficking, secretion, and vesicular transport  type IV pilus secretin PilQ | HofQ  Type II secretory pathway, component HofQ [Intracellular trafficking, secretion, and vesicular transport] | 100% | 77.32 |
| 4 | WP_003110659.1 | Extracellular (9.64) | Outside | 0.7693  (Probable ANTIGEN) | 0.946298 | -0.03 | Non-Allergen | PROBABLE ALLERGEN | Virulence factors (2.4049526) | Function unknown  Aegerolysin | Aegerolysin | 93.90% | 14.54 |
| 5 | WP_034064978.1 | Outer Membrane (10.00) | Outside | 0.6872   (Probable ANTIGEN) | 0.665470 | 0.15 | Non-Allergen | PROBABLE NON-ALLERGEN | Virulence factors  (-1.3316378) | Inorganic ion transport and metabolism  Receptor | CirA  Outer membrane receptor proteins | 94.16% | 84.65 |
| 6 | WP_125033182.1 | Outer Membrane (10.00) | Outside | 0.6754   (Probable ANTIGEN) | 0.795477 | 0.19 | Non-Allergen | PROBABLE NON-ALLERGEN | Virulence factors  (1.7212421) | Inorganic ion transport and metabolism  COG1629 Outer membrane receptor proteins, mostly Fe transport | CirA  Outer membrane receptor proteins | 93.40% | 76.73 |
| 7 | WP_003089339.1 | Outer Membrane (10.00) | Outside | 0.7615   (Probable ANTIGEN) | 0.777075 | 0.02 | Non-Allergen | PROBABLE NON-ALLERGEN | Virulence factors  (2.0825995) | Inorganic ion transport and metabolism  COG1629 Outer membrane receptor proteins, mostly Fe transport | ligand_gated_channel  TonB dependent/Ligand-Gated channels  CirA  Outer membrane receptor proteins, mostly Fe transport  TonB-siderophore  TonB-dependent siderophore receptor | 90.60% | 77.61 |
| 8 | WP_088365172.1 | Outer  Membrane (10.00) | Outside | 0.6488  (Probable ANTIGEN) | 0.576249 | 0.11 | Non-Allergen | PROBABLE NON-ALLERGEN | Virulence factors  (0.5862264) | Inorganic ion transport and metabolism  Receptor | ligand_gated_channel  TonB dependent/Ligand-Gated channels  CirA  Outer membrane receptor proteins, mostly Fe transport  TonB-siderophore  TonB-dependent siderophore receptor | 92.63% | 108.08 |
| 9 | WP_250870963.1 | Outer Membrane (10.00) | Outside | 0.6699   (Probable ANTIGEN) | 0.472401 | 0.18 | Non-Allergen | PROBABLE NON-ALLERGEN | Cellular process (0.1769566) | Inorganic ion transport and metabolism  siderophore transport | ligand_gated_channel  TonB dependent/Ligand-Gated channels | 94.92% | 81.22 |
| 10 | WP_128552673.1 | Outer Membrane (10.00) | Outside | 0.6552   (Probable ANTIGEN) | 0.833176 | 0.21 | Non-Allergen | PROBABLE NON-ALLERGEN | Cellular process (-0.431502) | Inorganic ion transport and metabolism  11COG4772 Outer membrane receptor for Fe3 -dicitrate | FecA  Outer membrane receptor for Fe3+-dicitrate  ligand_gated_channel  TonB dependent/Ligand-Gated channels  TonB-siderophore  TonB-dependent siderophore receptor | 94.41% | 79.38 |
| 11 | WP_132667381.1 | Outer Membrane (10.00) | Outside | 0.6306  (Probable ANTIGEN) | 0.776848 | -0.4 | Non-Allergen | PROBABLE NON-ALLERGEN | Cellular process (-2.757785) | Inorganic ion transport and metabolism  COG4772 Outer membrane receptor for Fe3 -dicitrate | FecA  Outer membrane receptor for Fe3+-dicitrate | 94.67% | 85.44 |
| 12 | WP_003091018.1 | Outer Membrane (10.00) | Outside | 0.6773   (Probable ANTIGEN) | 0.730808 | -0.46 | Non-Allergen | PROBABLE NON-ALLERGEN | Cellular process  (1.1087357) | Inorganic ion transport and metabolism  siderophore transport | Ligand_gated_channel  TonB dependent/Ligand-Gated channels | 100% | 80.39 |
| 13 | WP_257662817.1 | Outer Membrane (10.00) | outside | 0.6102 (Probable ANTIGEN) | 0.581419 | -0.45 | Non-Allergen | PROBABLE NON-ALLERGEN | Virulence factors  (0.8178421) | Inorganic ion transport and metabolism  COG1629 Outer membrane receptor proteins, mostly Fe transport | Ligand_gated_channel  TonB dependent/Ligand-Gated channels  OM channels superfamily | 75.1% | 90.48 |
| 14 | WP_121286032.1 | Outer  Membrane (10.00) | Outside | 0.6383   (Probable ANTIGEN) | 0.814213 | 0.15 | Non-Allergen | PROBABLE NON-ALLERGEN | Virulence factors  (-0.015091) | Inorganic ion transport and metabolism  TonB-dependent siderophore receptor | Ligand_gated_channel  OM channels superfamily  TonB dependent/Ligand-Gated channels  STN  Secretin and TonB N terminus short domain | 93.65% | 86.58 |
| 15 | WP_033895660.1 | Outer Membrane (10.00) | Outside | 0.7240 (Probable ANTIGEN) | 0.612743 | 0.06 | Non-Allergen | PROBABLE NON-ALLERGEN | Virulence factors  (2.1629536) | Inorganic ion transport and metabolism  siderophore transport | Fiu  Outer membrane receptor for monomeric catechols  ligand_gated_channel  TonB dependent/Ligand-Gated channels | 94.41 | 80.78 |
| 16 | WP_219104305.1 | Outer Membrane 10.00 | Outside | 0.6422  (Probable ANTIGEN) | 0.914951 | -0.09 | Non-Allergen | PROBABLE NON-ALLERGEN | Virulence factors  (1.9091478) | Inorganic ion transport and metabolism  siderophore transport | Fiu  Outer membrane receptor for monomeric catechols  ligand_gated_channel  TonB dependent/Ligand-Gated channels | 94.41 | 83.59 |
| 17 | HBP5664436.1 | Outer Membrane (9.93) | Outside | 0.6136   (Probable ANTIGEN) | 0.744738 | -0.49 | Non-Allergen | PROBABLE NON-ALLERGEN | Cellular process  (0.4899141) | Inorganic ion transport and metabolism  Receptor | TonB-hemin superfamily  TonB-dependent heme/hemoglobin receptor family protein  STN  Secretin and TonB N terminus short domain | 94.67% | 95.19 |
| 18 | WP_126468965.1 | Outer Membrane (10.00) | Outside | 0.6716   (Probable ANTIGEN) | 0.823697 | 0.16 | Non-Allergen | PROBABLE NON-ALLERGEN | Cellular process  (0.222303) | Inorganic ion transport and metabolism  Receptor | TonB-hemlactrns super family  TonB-dependent hemoglobin/transferrin/lactoferrin receptor family protein | 100% | 84.69 |
| 19 | WP_114012797.1 | Outer Membrane (10.00) | Outside | 0.6037   (Probable ANTIGEN) | 0.710245 | -0.44 | Non-Allergen | PROBABLE NON-ALLERGEN | Virulence factors  (-0.204377) | Inorganic ion transport and metabolism  COG1629 Outer membrane receptor proteins, mostly Fe transport | TonB-hemin superfamily  TonB-dependent heme/hemoglobin receptor family protein | 93.65% | 108.13 |
| 20 | WP_003084084.1 | Outer Membrane (10.00) | Outside | 0.664 7 (Probable ANTIGEN) | 0.430032 | 0.19 | Non-Allergen | PROBABLE NON-ALLERGEN | Cellular process  (0.3429235) | Function unknown  outer membrane (Porin) | OprD  outer membrane porin | 100% | 45.56 |
| 21 | WP_003093566.1 | Outer Membrane (10.00) | Outside | 0.7731  (Probable ANTIGEN) | 0.702169 | 0.1 | Non-Allergen | PROBABLE NON-ALLERGEN | Virulence factors (1.5489836) | Function unknown  outer membrane porin, OprD family | OprD  Outer membrane porin, OprD family | 93.65% | 47.73 |
| 22 | WP_003090815.1 | Outer Membrane (10.00) | Outside | 0.7542   (Probable ANTIGEN) | 0.233645 | 0.06 | Non-Allergen | PROBABLE NON-ALLERGEN | Cellular process  (-0.0549665) | Function unknown  outer membrane porin, OprD family | OprD  Outer membrane porin | 100% | 46.89 |
| 23 | WP_003090563.1 | Outer Membrane (10.00) | Outside | 0.7847  (Probable ANTIGEN) | 0.314828 | 0.01 | Non-Allergen | PROBABLE NON-ALLERGEN | Virulence factors  (1.8009127) | Function unknown  outer membrane porin, OprD family | OprD  Outer membrane porin | 94.92% | 47.24 |
| 24 | WP_003089648.1 | Outer Membrane (10.00) | Outside | 0.8284   (Probable ANTIGEN) | 0.367256 | 0.13 | Non-Allergen | PROBABLE NON-ALLERGEN | Cellular process  (0.7723714) | Function unknown  outer membrane (Porin) | OprD  Outer membrane porin, OprD family | 93.90% | 51.27 |
| 25 | WP_023103473.1 | Outer Membrane (10.00) | Outside | 0.7378   (Probable ANTIGEN) | 0.811841 | 0.12 | Non-Allergen | PROBABLE NON-ALLERGEN | Metabolism Molecule  (-1.4943536) | Function unknown  wide pore channel activity | OprD  Outer membrane porin, OprD family; This family includes outer membrane proteins related to OprD. OprD has been described as a serine-type peptidase. | 100% | 46.98 |
| 26 | WP_073660276.1 | Outer Membrane (10.00) | Outside | 0.7514  (Probable ANTIGEN) | 0.859334 | 0.23 | Non-Allergen | PROBABLE NON-ALLERGEN | Cellular process  (-0.864663) | Function unknown  outer membrane (Porin) | OprD  Outer membrane porin, OprD family; This family includes outer membrane proteins related to OprD. OprD has been described as a serine-type peptidase | 94.92% | 53.14 |
| 27 | WP_117407697.1 | Outer Membrane (10.00) | Outside | 0.6409   (Probable ANTIGEN) | 0.745307 | 0.18 | Non-Allergen | PROBABLE NON-ALLERGEN | Cellular process  (-0.245541) | Cell wall/membrane/envelope biogenesis  lipopolysaccharide transport | LptD  LPS assembly outer membrane protein LptD | 94.92% | 105.13 |
| 28 | WP_023098436.1 | Outer Membrane (9.49) | Outside | 0.6393   (Probable ANTIGEN) | 0.493775 | 0.04 | Non-Allergen | PROBABLE NON-ALLERGEN | Information and Storage  (-1.892982) | Cell cycle control, cell division, chromosome partitioning  tail tape measure protein | HI1514  Phage tail tape-measure protein controls the tail length | 77.41% | 77.61 |
| 29 | WP_014604038.1 | Outer  Membrane 10.00 | Outside | 0.6260  (Probable ANTIGEN) | 0.579191 | -0.45 | Non-Allergen | PROBABLE NON-ALLERGEN | Cellular process  (-1.2827296) | Cell wall/membrane/envelope biogenesis, Intracellular trafficking, secretion, and vesicular transport  CyaE is necessary for the transport of calmodulin-sensitive adenylate cyclase-hemolysin (cytolysin) | TolC  Outer membrane protein TolC | 94.41 | 50.62 |
| 30 | WP_134227903.1 | Outer Membrane (10.00) | Outside | 0.6502   (Probable ANTIGEN) | 0.631637 | -0.47 | Non-Allergen | PROBABLE NON-ALLERGEN | Cellular process  (-2.9244884) | Cell wall/membrane/envelope biogenesis  RND efflux system, outer membrane lipoprotein | TolC  Outer membrane protein TolC [Cell wall/membrane/envelope biogenesis] | 93.90% | 51.17 |
| 31 | WP_074202871.1 | Outer Membrane 10.00 | Outside | 0.6589   (Probable ANTIGEN) | 0.759357 | -0.43 | Non-Allergen | PROBABLE NON-ALLERGEN | Cellular process  (-3.9717782) | Cell wall/membrane/envelope biogenesis, Intracellular trafficking, secretion, and vesicular transport  RND efflux system, outer membrane lipoprotein | Outer_NodT  efflux transporter, outer membrane factor (OMF) lipoprotein  TolC  Outer membrane protein TolC [Cell wall/membrane/envelope biogenesis] | 94.92% | 52.77 |
| 32 | WP_126559318.1 | Outer Membrane (10.00) | Outside | 0.6305  (Probable ANTIGEN) | 0.766013 | 0.07 | Non-Allergen | PROBABLE ALLERGEN | Virulence factors  (0.6037067) | Cell wall/membrane/envelope biogenesis  RND efflux system, outer membrane lipoprotein | outer_NodT superfamily  efflux transporter, outer membrane factor (OMF) lipoprotein, NodT family | 100% | 53.86 |
| 33 | WP_003093373.1 | Outer Membrane (10.00) | Outside | 0.6802  (Probable ANTIGEN) | 0.749065 | -0.41 | Non-Allergen | PROBABLE NON-ALLERGEN | Cellular process  (-1.2486563) | Eukaryota | FimD  Outer membrane usher protein FimD/PapC | 100% | 91.17 |
| 34 | WP_003086235.1 | Outer Membrane (10.00) | Outside | 0.6642   (Probable ANTIGEN) | 0.352499 | -0.46 | Non-Allergen | PROBABLE NON-ALLERGEN | Cellular process  (-5.7196892) | Cell motility, Intracellular trafficking, secretion, and vesicular transport  fimbrial usher porin activity | FimD  Outer membrane usher protein FimD/PapC [Cell motility, Extracellular structures] | 93.14% | 91.45 |
| 35 | WP_003094977.1 | Outer Membrane (10.00) | Outside | 0.6927   (Probable ANTIGEN) | 0.478804 | 0.28 | Non-Allergen | PROBABLE NON-ALLERGEN | Metabolism Molecule  (-3.4498568) | - | FimD  Outer membrane usher protein FimD/PapC [Cell motility, Extracellular structures] | 94.16% | 86.1 |
| 36 | WP_034048375.1 | Extracellular (9.71) | Outside | 0.6178   (Probable ANTIGEN) | 0.602667 | 0.04 | Non-Allergen | PROBABLE NON-ALLERGEN | Virulence factors  (0.7749407) | Inorganic ion transport and metabolism  PhoD-like phosphatase | PhoD  Phosphodiesterase/alkaline phosphatase D | 100% | 58.8 |
| 37 | WP_234592198.1 | Outer Membrane (10.00) | outside | 0.6442 (Probable ANTIGEN) | 0.942559 | -0.41 | Non-Allergen | PROBABLE NON-ALLERGEN | Cellular process (-0.745164) | Cell wall/membrane/envelope biogenesis  Belongs to the ompA family | OmpA  Outer membrane protein OmpA and related peptidoglycan-associated (lipo)proteins | 100% | 28.51 |
| 38 | WP_003087843.1 | Outer  Membrane (10.00) | Outside | 0.8044  (Probable ANTIGEN) | 0.949137 | 0.14 | Non-Allergen | PROBABLE NON-ALLERGEN | Cellular process  (-3.4169107) | Cell wall/membrane/envelope biogenesis  Belongs to the ompA family | OprF  OprF membrane domain  OmpA_C-like  Peptidoglycan binding domains similar to the C-terminal domain of outer-membrane protein OmpA | 100% | 37.63 |
| 39 | WP_257654140.1 | Outer  Membrane (9.93) | Outside | 0.6615   (Probable ANTIGEN) | 0.762971 | -0.31 | Non-Allergen | PROBABLE NON-ALLERGEN | Cellular process  (-0.522729) | Cell motility  Belongs to the ompA family | MotY_N  MotY N-terminal domain  OmpA  Outer membrane protein OmpA and related peptidoglycan-associated (lipo)proteins | 100% | 35.12 |
| 40 | WP_033942568.1 | Outer  Membrane (10.00) | Outside | 0.7113  (Probable ANTIGEN) | 0.812473 | 0.25 | Non-Allergen | PROBABLE NON-ALLERGEN | Metabolism Molecule  (-2.2439658) | Cell wall/membrane/envelope biogenesis  Belongs to the ompA family | OmpA  Outer membrane protein OmpA and related peptidoglycan-associated (lipo)proteins | 100% | 21.74 |
| 41 | WP_058016985.1 | Outer  Membrane (9.45) | Outside | 0.8944  (Probable ANTIGEN) | 0.951126 | 0.06 | Non-Allergen | PROBABLE NON-ALLERGEN | Metabolism Molecule  (-1.9051873) | Cell wall/membrane/envelope biogenesis  Belongs to the ompA family | OmpA  Outer membrane protein OmpA and related peptidoglycan-associated (lipo)proteins | 100% | 24.71 |
| 42 | WP_128733447.1 | Outer  Membrane (10.00) | Outside | 1.0550  (Probable ANTIGEN) | 0.772268 | 0.01 | Non-Allergen | PROBABLE NON-ALLERGEN | Metabolism Molecule  (-1.9911931) | Cell wall/membrane/envelope biogenesis  Belongs to the ompA family | Pal_lipo  peptidoglycan-associated lipoprotein; Members of this protein are Pal | 100% | 17.92 |
| 43 | WP_222173885.1 | Outer  Membrane (10.00) | Outside | 0.6951   (Probable ANTIGEN) | 0.860814 | 0.15 | Non-Allergen | PROBABLE NON-ALLERGEN | Metabolism Molecule  (0.3948993) | Function unknown  Alginate regulatory protein | Alginate_exp  Alginate export | 100% | 54.42 |
| 44 | WP_003122437.1 | Outer  Membrane (10.00) | Outside | 0.7121  (Probable ANTIGEN) | 0.898399 | -0.36 | Non-Allergen | PROBABLE NON-ALLERGEN | Cellular process  (-1.8825472) | Inorganic ion transport and metabolism  Not Available | OprP  Phosphate-selective porin  Porin_O_P  Phosphate-selective porin O and P | 100% | 48.23 |
| 45 | WP_003089495.1 | Extracellular (10.00) | Outside | 0.8968   (Probable ANTIGEN) | 0.500000 | -0.22 | Non-Allergen | PROBABLE ALLERGEN | Cellular process  (-0.978184) | Function unknown  Type VI secretion system effector, Hcp | Hcp  Type VI protein secretion system component Hcp (secreted cytotoxin) | 92.63% | 17.63 |
| 46 | WP_003117829.1 | Extracellular (9.71) | Outside | 0.6375  (Probable ANTIGEN) | 0.831568 | -0.4 | Non-Allergen | PROBABLE NON-ALLERGEN | Metabolism Molecule  (0.2215219) | Carbohydrate transport and metabolism  Belongs to the glycosyl hydrolase 18 family | Chi1  Chitinase [Carbohydrate transport and metabolism] | 89.59% | 53.04 |
| 47 | WP_015502915.1 | Outer  Membrane (10.00) | Outside | 0.6402   (Probable ANTIGEN) | 0.910958 | -0.03 | Non-Allergen | PROBABLE NON-ALLERGEN | Metabolism Molecule  (-1.0945509) | Cell wall/membrane/envelope biogenesis  wide pore channel activity | OprB  Carbohydrate-selective porin OprB [Cell wall/membrane/envelope biogenesis] | 94.67% | 50.54 |
| 48 | WP_078457105.1 | Extracellular (10.00) | Outside | 0.6771  (Probable ANTIGEN) | 0.902297 | 0.21 | Non-Allergen | PROBABLE NON-ALLERGEN | Metabolism Molecule  (-1.1247692) | Cell motility, Intracellular trafficking, secretion, and vesicular transport  Fimbrial protein | FimA  Pilin (type 1 fimbria component protein) [Cell motility] | 90.10% | 18.15 |
| 49 | WP_034004502.1 | Extracellular 9.72 | Outside | 1.2357  (Probable ANTIGEN) | 0.893886 | -0.28 | Non-Allergen | PROBABLE NON-ALLERGEN | Metabolism Molecule  (-0.2481224) | Cell motility, Intracellular trafficking, secretion, and vesicular transport  Fimbrial protein | FimA  Pilin (type 1 fimbria component protein) [Cell motility] | 92.38% | 20.19 |
| 50 | WP_257318590.1 | Outer  Membrane (9.52) | Outside | 0.7118  (Probable ANTIGEN) | 0.893886 | 0.11 | Non-Allergen | PROBABLE NON-ALLERGEN | Metabolism Molecule  (1.2491111) | Cell wall/membrane/envelope biogenesis  Has lipid A 3-O-deacylase activity. Hydrolyzes the ester bond at the 3 positions of lipid A, a bioactive component of lipopolysaccharide (LPS), thereby releasing the primary fatty acyl moiety | Phenol_MetA_deg  Putative MetA-pathway of phenol degradation  ZIP_TSC22D-like superfamily  leucine zipper found in the TSC22 domain leucine zipper transcription factors  YbgF superfamily  Periplasmic TolA-binding protein (function unknown) [General function prediction only] | 94.16% | 45.71 |
| 51 | HCE9289693.1 | Extracellular (9.71) | Outside | 0.7718  (Probable ANTIGEN) | 0.825230 | 0.05 | Non-Allergen | PROBABLE NON-ALLERGEN | \|  \| \| --- \|   Virulence factors  (1.8649548) | Function unknown  Yop proteins translocation protein P | Type_III_yscP  type III secretion system needle length determinant  PHA03247 superfamily  large tegument protein UL36 | 88.8% | 38.76 |
| 52 | WP_126558827.1 | Outer Membrane (10.00) | Outside | 0.7986  (Probable ANTIGEN) | 0.915193 | -0.4 | Non-Allergen | PROBABLE NON-ALLERGEN | Virulence factors  (0.34028568) | Lipid transport and metabolism  long-chain fatty acid transporting porin activity | Toluene_X  Outer membrane protein transport protein (OMPP1/FadL/TodX) | 94.92% | 45.56 |
| 53 | WP_004364348.1 | Outer Membrane (9.71) | Outside | 0.6870   (Probable ANTIGEN) | 0.874463 | 0.11 | Non-Allergen | PROBABLE NON-ALLERGEN | Cellular process  (0.4128236) | Lipid transport and metabolism  long-chain fatty acid transporting porin activity | Toluene_X  Outer membrane protein transport protein (OMPP1/FadL/TodX) | 93.40% | 49.72 |
| 54 | WP_003086411.1 | Extracellular (9.96) | Outside | 0.6370   (Probable ANTIGEN) | 0.937568 | 0.14 | Non-Allergen | PROBABLE NON-ALLERGEN | Cellular process  (-0.5805786) | Cell motility  flagellar hook-associated protein | flgK superfamily  flagellar hook-associated protein FlgK | 94.41% | 71.43 |
| 55 | WP_003086407.1 | Outer Membrane (9.92) | Outside | 0.7306   (Probable ANTIGEN) | 0.938748 | 0.23 | Non-Allergen | PROBABLE NON-ALLERGEN | Cellular process  (-0.87481724) | Cell motility  Assembles around the rod form the L-ring and probably protects the motor basal body from shearing forces during rotation | FlgH  flagellar basal body L-ring protein FlgH | 100% | 24.46 |
| 56 | WP_003086405.1 | Extracellular (10.00) | Outside | 0.6573   (Probable ANTIGEN) | 0.942130 | -0.26 | Non-Allergen | PROBABLE NON-ALLERGEN | Virulence factors  (0.76243979) | Cell motility  bacterial-type flagellum-dependent cell motility | FlgE  flagellar hook protein FlgE | 100% | 48.32 |
| 57 | WP_031690149.1 | Extracellular (10.00) | Outside | 0.6798  (Probable ANTIGEN) | 0.909418 | 0.3 | Non-Allergen | PROBABLE ALLERGEN | Virulence factors  (2.1503218) | Cell motility  Required for flagellar hook formation. May act as a scaffolding protein | FlgD  flagellar hook assembly protein FlgD | 100% | 24.85 |
| 58 | WP_125908912.1 | Outer Membrane (10.00) | Outside | 0.8026   (Probable ANTIGEN) | 0.809888 | -0.21 | Non-Allergen | PROBABLE ALLERGEN | Metabolism Molecule  (-1.01542) | Cell wall/membrane/envelope biogenesis  Outer membrane lipoprotein | SlyB  Outer membrane lipoprotein SlyB [Cell wall/membrane/envelope biogenesis] | 100% | 15.64 |
| 59 | WP_132866313.1 | Outer  Membrane (9.49) | Outside | 0.6149   (Probable ANTIGEN) | 0.821847 | -0.25 | Non-Allergen | PROBABLE NON-ALLERGEN | Cellular process  (0.51010563) | Cell wall/membrane/envelope biogenesis  Gram-negative-bacterium-type cell outer membrane assembly | NlpB  Uncharacterized lipoprotein, NlpB/DapX family [Function unknown] | 100% | 43.05 |
| 60 | WP_003086036.1 | Outer  Membrane (10.00) | Outside | 0.7192   (Probable ANTIGEN) | 0.943290 | -0.41 | Non-Allergen | PROBABLE ALLERGEN | Cellular process  (-4.4030116) | Inorganic ion transport and metabolism  Receptor | PRK13528  outer membrane receptor FepA | 100% | 80.97 |
| 61 | WP_132649933.1 | Outer Membrane (10.00) | Outside | 0.6427   (Probable ANTIGEN) | 0.568231 | -0.35 | Non-Allergen | PROBABLE NON-ALLERGEN | Virulence factors  (0.4441779) | Inorganic ion transport and metabolism  siderophore transport | PRK10044 superfamily  ferrichrome outer membrane transporter | 94.67% | 79.09 |
| 62 | WP_073664689.1 | Outer  Membrane (9.52) | Outside | 0.6073  (Probable ANTIGEN) | 0.861689 | 0.02 | Non-Allergen | PROBABLE NON-ALLERGEN | Cellular process  (-1.1284609) | Inorganic ion transport and metabolism  Putative porin | Porin_5  Putative porin | 94.41% | 61.93 |
| 63 | WP_134281237.1 | Outer  Membrane (10.00) | Outside | 0.6319  (Probable ANTIGEN) | 0.898028 | -0.31 | Non-Allergen | PROBABLE NON-ALLERGEN | Cellular process  (0.47365419) | Inorganic ion transport and metabolism  Imelysin | IrpA  Uncharacterized iron-regulated protein  Imelysin_IrpA-like  Imelysin-like iron-regulated protein A-like  Peptidase_M75  Imelysin; The imelysin peptidase was first identified in Pseudomonas aeruginosa | 100% | 47.23 |
| 64 | WP_003121016.1 | Extracellular (9.64) | Outside | 0.6190  (Probable ANTIGEN) | 0.890285 | 0.24 | Non-Allergen | PROBABLE NON-ALLERGEN | Cellular process  (1.0647564) | Function unknown  Not Available | Phage_T7_tail superfamily  Phage T7 tail fibre protein | 100% | 37.87 |
| 65 | WP_003095996.1 | Outer  Membrane (10.00) | Outside | 0.6755   (Probable ANTIGEN) | 0.755557 | -0.36 | Non-Allergen | PROBABLE NON-ALLERGEN | Virulence factors  (0.7448105) | Lipid transport and metabolism  lipase activity | COG3240  Phospholipase/lecithinase/hemolysin [Lipid transport and metabolism, General function prediction only]  Autotransporter  Secretion of protein products occurs by a number of different pathways in bacteria | 100% | 69.57 |
| 66 | WP_096078616.1 | Outer  Membrane (9.93) | Outside | 0.7458  (Probable ANTIGEN) | 0.865702 | -0.3 | Non-Allergen | PROBABLE NON-ALLERGEN | Cellular process  (0.27926627) | Cell wall/membrane/envelope biogenesis  the nucleoside-specific channel forming porin activity | Tsx  Nucleoside-specific outer membrane channel protein Tsx [Cell wall/membrane/envelope biogenesis] | 94.41% | 31.4 |
| 67 | WP_134302653.1 | Outer  Membrane (10.00) | Outside | 0.6830   (Probable ANTIGEN) | 0.929998 | -0.49 | Non-Allergen | PROBABLE NON-ALLERGEN | Virulence factors  (0.49286453) | Cell motility, Intracellular trafficking, secretion, and vesicular transport  general secretion pathway protein D | type_II_gspD  type II secretion system protein D  PulD  Type II secretory pathway component GspD/PulD (secretin) | 94.41 | 83.85 |
| 68 | WP_003094820.1 | Extracellular (10.00) | Outside | 0.8037   (Probable ANTIGEN) | 0.899950 | 0.29 | Non-Allergen | PROBABLE NON-ALLERGEN | Cellular process  (-0.045675783) | Function unknown  Not Available | No conserved domains have been identified for these query sequences | 93.65% | 16.16 |
| 69 | WP_134309249.1 | Outer  Membrane (10.00) | Outside | 0.9146   (Probable ANTIGEN) | 0.340896 | 0.17 | Non-Allergen | PROBABLE ALLERGEN | Cellular process  (-1.1657181) | Function unknown  Alanine-zipper, major outer membrane lipoprotein | No conserved domains have been identified for these query sequences | 100% | 8.83 |
| 70 | WP_128722813.1 | Extracellular (9.65) | Outside | 0.9235   (Probable ANTIGEN) | 0.930133 | -0.41 | Non-Allergen | PROBABLE NON-ALLERGEN | Cellular process  (0.021552418) | Function unknown  Not Available | No conserved domains have been identified for these query sequences | 93.40% | 15.91 |
| 71 | WP_128568932.1 | Extracellular (9.65) | Outside | 0.7883  (Probable ANTIGEN) |  | 0.26 | Non-Allergen | PROBABLE NON-ALLERGEN | Cellular process  (0.090746726) | Function unknown  Not Available | No conserved domains have been identified for these query sequences | 93.14% | 19.07 |
| 72 | WP_003088312.1 | Extracellular (9.65) | Outside | 0.7320   (Probable ANTIGEN) | 0.926922 | 0.26 | Non-Allergen | PROBABLE NON-ALLERGEN | Metabolism Molecule  (-1.5019752) | Function unknown  Not Available | No conserved domains have been identified for these query sequences | 93.40% | 25.62 |
