# Supplementary material for: Defeating a superbug: A breakthrough in vaccine design against multidrug-resistant Pseudomonas aeruginosa using reverse vaccinology
Source: PLoS One. 2023 Aug 3;18(8):e0289609. doi: 10.1371/journal.pone.0289609 (PMC10399887; doi:10.1371/journal.pone.0289609)
Supplement: S2 Table — (DOCX) [file pone.0289609.s003.docx]

**S3 Table.** Evaluation of linear B-cell epitopes and designing different multi-epitope proteins against *P. aeruginosa* 24Pae112.

| **Protein name**  **(Accession number)** | **Protein**  **length**  **(aa)** | **Conservation of linear B-cell epitopes**   1  2  3  4  5  6  7  8  9  **Variable Average Conserved** | **Homology to the human proteome** | **Start** | **End** | **Location** | **VaxiJen**  **antigenicity**  **Cut off: (0.4)** |
| --- | --- | --- | --- | --- | --- | --- | --- |
| WP_125941151.1  (TonB-dependent receptor) | 953 | **PEEIGGKSSNN** | - | 426 | 436 | Exposed | ANTIGEN  1.84 |
|  |  | **YNVSKASSSGG** | - | 579 | 589 | Exposed | ANTIGEN  1.53 |
|  |  | **QADANG YYPAQKDYYG CEKGVDGAC** | - | 663 | 687 | Exposed | ANTIGEN  1.06 |
| WP_024947839.1  (Transporter) | 397 | **GSTSQITEKSVTGD** | - | 178 | 191 | Exposed | ANTIGEN  1.62 |
| WP_019681707.1  (OprD family porin) | 447 | **YTAPG NTRGNS** | - | 421 | 431 | Exposed | ANTIGEN  1.58 |

>Chimeric_ FliC

MALTVNTNIASLNTQRNLNNSSASLNTSLQRLSTGSRINSAKDDAAGLQIANRLTSQVNGLNVATKNANDGISLAQTAEGALQQSTNILQRMRDLSLQSANGSNSDSERTALNGEVKQLQKELDRISNTTTFGGRKLLDGSFGVASFQVGSAANEIISVGIDEMSAESLNGTEAAAKQADANGYYPAQKDYYGCEKGVDGACGPGPGGSTSQITEKSVTGDGPGPGPEEIGGKSSNNGPGPGYTAPGNTRGNSGPGPGYNVSKASSSGGEAAAKTDTGSTGAGTAAGTTTFTEANDTVAKIDISTAKGAQSAVLVIDEAIKQIDAQRADLGAVQNRFDNTINNLKNIGENVSAARGRIEDTDFAAETANLTKNQVLQQAGTAILAQANQLPQSVLSLLR

| No. | Epitopes | Antigenicity |
| --- | --- | --- |
| 1 | EAAAKQADANGYYPAQKDYYGCEKGVDGACGPGPGPEEIGGKSSNNGPGPGYTAPGNTRGNSGPGPGYNVSKASSSGGGPGPGGSTSQITEKSVTGDEAAAK | 1.6907 |
| 2 | EAAAKQADANGYYPAQKDYYGCEKGVDGACGPGPGYTAPGNTRGNSGPGPGPEEIGGKSSNNGPGPGYNVSKASSSGGGPGPGGSTSQITEKSVTGDEAAAK | 1.6907 |
| 3 | EAAAKQADANGYYPAQKDYYGCEKGVDGACGPGPGYNVSKASSSGGGPGPGPEEIGGKSSNNGPGPGYTAPGNTRGNSGPGPGGSTSQITEKSVTGDEAAAK | 1.6907 |
| 4 | EAAAKQADANGYYPAQKDYYGCEKGVDGACGPGPGGSTSQITEKSVTGDGPGPGPEEIGGKSSNNGPGPGYTAPGNTRGNSGPGPGYNVSKASSSGGEAAAK | 1.6946 |
| 5 | EAAAKPEEIGGKSSNNGPGPGYTAPGNTRGNSGPGPGQADANGYYPAQKDYYGCEKGVDGACGPGPGYNVSKASSSGGGPGPGGSTSQITEKSVTGDEAAAK | 1.6534 |
| 6 | EAAAKPEEIGGKSSNNGPGPGYNVSKASSSGGGPGPGYTAPGNTRGNSGPGPGGSTSQITEKSVTGDGPGPGQADANGYYPAQKDYYGCEKGVDGACEAAAK | 1.6157 |
| 7 | EAAAKPEEIGGKSSNNGPGPGGSTSQITEKSVTGDGPGPGYTAPGNTRGNSGPGPGYNVSKASSSGGGPGPGQADANGYYPAQKDYYGCEKGVDGACEAAAK | 1.6157 |
| 8 | EAAAKPEEIGGKSSNNGPGPGQADANGYYPAQKDYYGCEKGVDGACGPGPGYTAPGNTRGNSGPGPGYNVSKASSSGGGPGPGGSTSQITEKSVTGDEAAAK | 1.6534 |
| 9 | EAAAKYTAPGNTRGNSGPGPGPEEIGGKSSNNGPGPGQADANGYYPAQKDYYGCEKGVDGACGPGPGYNVSKASSSGGGPGPGGSTSQITEKSVTGDEAAAK | 1.6179 |
| 10 | EAAAKYTAPGNTRGNSGPGPGYNVSKASSSGGGPGPGPEEIGGKSSNNGPGPGGSTSQITEKSVTGDGPGPGQADANGYYPAQKDYYGCEKGVDGACEAAAK | 1.5802 |
| 11 | EAAAKYTAPGNTRGNSGPGPGGSTSQITEKSVTGDGPGPGPEEIGGKSSNNGPGPGYNVSKASSSGGGPGPGQADANGYYPAQKDYYGCEKGVDGACEAAAK | 1.5802 |
| 12 | EAAAKYTAPGNTRGNSGPGPGQADANGYYPAQKDYYGCEKGVDGACGPGPGPEEIGGKSSNNGPGPGYNVSKASSSGGGPGPGGSTSQITEKSVTGDEAAAK | 1.6179 |
| 13 | EAAAKYNVSKASSSGGGPGPGPEEIGGKSSNNGPGPGQADANGYYPAQKDYYGCEKGVDGACGPGPGYTAPGNTRGNSGPGPGGSTSQITEKSVTGDEAAAK | 1.6150 |
| 14 | EAAAKYNVSKASSSGGGPGPGYTAPGNTRGNSGPGPGPEEIGGKSSNNGPGPGGSTSQITEKSVTGDGPGPGQADANGYYPAQKDYYGCEKGVDGACEAAAK | 1.5773 |
| 15 | EAAAKYNVSKASSSGGGPGPGGSTSQITEKSVTGDGPGPGPEEIGGKSSNNGPGPGYTAPGNTRGNSGPGPGQADANGYYPAQKDYYGCEKGVDGACEAAAK | 1.5773 |
| 16 | EAAAKYNVSKASSSGGGPGPGQADANGYYPAQKDYYGCEKGVDGACGPGPGPEEIGGKSSNNGPGPGYTAPGNTRGNSGPGPGGSTSQITEKSVTGDEAAAK | 1.6150 |
| 17 | EAAAKGSTSQITEKSVTGDGPGPGPEEIGGKSSNNGPGPGQADANGYYPAQKDYYGCEKGVDGACGPGPGYTAPGNTRGNSGPGPGYNVSKASSSGGEAAAK | 1.6286 |
| 18 | EAAAKGSTSQITEKSVTGDGPGPGYTAPGNTRGNSGPGPGPEEIGGKSSNNGPGPGYNVSKASSSGGGPGPGQADANGYYPAQKDYYGCEKGVDGACEAAAK | 1.5870 |
| 19 | EAAAKGSTSQITEKSVTGDGPGPGYNVSKASSSGGGPGPGPEEIGGKSSNNGPGPGYTAPGNTRGNSGPGPGQADANGYYPAQKDYYGCEKGVDGACEAAAK | 1.5870 |
| 20 | EAAAKGSTSQITEKSVTGDGPGPGQADANGYYPAQKDYYGCEKGVDGACGPGPGPEEIGGKSSNNGPGPGYTAPGNTRGNSGPGPGYNVSKASSSGGEAAAK | 1.6286 |

>Chimeric cell-wall associated transporter:

GAPAPSVENIYQDASGFFGGGTFSLETGLTYSHYQADANGYYPAQKDYYGCEKGVDGACQIDADIWTLDLTGRYNWNQRWQVDINAPVVYRESTYQPEEIGGKSSNNKSVTGDPRLGDVSFGVAYKFLDESESTPDAVVSLRVKAPTGKDPYGIKLKQVPGNNNLNVPDDLPTGNGVWSITPGISLVKTVDPAVLFGSLSYTYNFEESFYTAPGNTRGNSGKVKLGNWFQLGVGVAFALNEKMSMSFSFSELISQKSKVKQYNVSKASSSGGANAGYFGLGMTYAVSNRFSIVPSLSIGGSTSQITEKSVTGDFTFGVKFPYYF

>Chimeric bacteriophage T7 tail protein

KNTTISISNAQALNIDTLLKATTTQADANGYYPAQKDYYGCEKGVDGACGAELTANNLQEVINLAGSLTRVSTIAAQAININTLLSAISTAGNSKSFSAEFGSTSQITEKSVTGDSDNLLRAVNAAGTNTSISVNTAQATNITALLQTIHAAGNTKTFSAEFNGAQLTSNNIQQALDAAGTRTSISVNTAQAVNISTLLALINSAKDTKKFSADFNGAQLTADNLQQAISAAAAGTSISVNTAQAANISILLQVINIAGNTKKFSANFPEEIGGKSSNNSNNIQQALRAAGSNTSISMNYNVSKASSSGGQSTLLELLDIASSSKQFQANYYTAPGNTRGNSPSNLQQIVSRAGASATVFISDAQGLPIANILTLISSAG
